# A novel long non‐coding RNA *LINC00524* facilitates invasion and metastasis through interaction with TDP43 in breast cancer

**DOI:** 10.1111/jcmm.18275

**Published:** 2024-04-03

**Authors:** Xianglin Sun, Wenfeng Li, Gang Li, Huan Yang, Zhenglin Jiang, Lihua Shen, Yuexiang Shen, Yifei Liu, Guohua Wang

**Affiliations:** ^1^ Department of Physiology and Hypoxic Biomedicine, Institute of Special Environmental Medicine Nantong University Nantong China; ^2^ Department of Pathology Affiliated Hospital of Nantong University Nantong China; ^3^ Juquan Community Health Service Center Shanghai China

**Keywords:** breast cancer, cancer metastasis, *LINC00524*, protein–RNA interactions, TDP43

## Abstract

Breast cancer (BC) remains a significant health concern worldwide, with metastasis being a primary contributor to patient mortality. While advances in understanding the disease's progression continue, the underlying mechanisms, particularly the roles of long non‐coding RNAs (lncRNAs), are not fully deciphered. In this study, we examined the influence of the lncRNA *LINC00524* on BC invasion and metastasis. Through meticulous analyses of TCGA and GEO data sets, we observed a conspicuous elevation of *LINC00524* expression in BC tissues. This increased expression correlated strongly with a poorer prognosis for BC patients. A detailed Gene Ontology analysis suggested that *LINC00524* likely exerts its effects through RNA‐binding proteins (RBPs) mechanisms. Experimentally, *LINC00524* was demonstrated to amplify BC cell migration, invasion and proliferation in vitro. Additionally, in vivo tests showed its potent role in promoting BC cell growth and metastasis. A pivotal discovery was *LINC00524*'s interaction with TDP43, which leads to the stabilization of TDP43 protein expression, an element associated with unfavourable BC outcomes. In essence, our comprehensive study illuminates how *LINC00524* accelerates BC invasion and metastasis by binding to TDP43, presenting potential avenues for therapeutic interventions.

## 1 INTRODUCTION

Cancer constitutes the primary cause of human mortality and presents an escalating global public health concern.^1^, ^2^ In both developed and developing countries, breast cancer (BC) incidence and mortality rates are still rising.^3^, ^4^ Notably, BC metastasis significantly contributes to heightened mortality among BC patients.^5^ Multi‐omics investigations have identified breast tumour heterogeneity as the principal factor responsible for metastasis, invasion and drug resistance in BC.^6^, ^7^ The advancement of contemporary biotechnology, encompassing bioinformatics and microarray studies, has facilitated extensive discussion and research on novel molecular indicators corresponding to the BC onset, progression, metastasis and prognosis.^8^, ^9^ Consequently, there is a pressing need to employ bioinformatics methodologies to screen for novel BC markers and offer innovative strategies for precision BC therapies.

Long non‐coding RNAs (lncRNAs) are RNA molecules typically longer than 200 nucleotides.^10^ Many lncRNAs exhibit explicit expression under specific conditions and tissues, which can contribute to the progression of numerous diseases, which includes cancer.^11^, ^12^, ^13^ In BC, lncRNAs are implicated in disease development, diagnosis, treatment and prognosis, and can influence BC cell proliferation, invasion, metastasis and drug resistance.^12^, ^14^, ^15^, ^16^, ^17^ lncRNAs mediate gene expression in BC through several mechanisms of action; besides their role as competing endogenous RNAs (ceRNAs).^18^, ^19^ lncRNAs predominantly interact with proteins to form lncRNA‐protein complexes (lncRNPs) that regulate target gene transcription and splicing, thereby controlling downstream gene expression and signalling pathways.^20^, ^21^, ^22^ Thus, comprehending the lncRNA–protein interaction mechanism in BC is crucial for establishing a robust foundation for lncRNAs as novel BC markers and therapeutic prognostic indicators.

TAR DNA‐binding protein 43 (TDP43; also referred to as TARDBP or TDP‐43) belongs to the family of heterogeneous nuclear ribonucleoprotein (hnRNP) splicing factors and can regulate various biological processes, including transcriptional repression, mRNA splicing, microRNA processing and mRNA stability.^23^, ^24^ Current research on TDP43 predominantly focuses on the pathogenesis of multiple forms of frontotemporal lobar degeneration and amyotrophic lateral sclerosis.^25^, ^26^ Nevertheless, the function of TDP43 in BC, particularly its potential involvement with lncRNAs, has garnered limited attention.^27^

In this research, we applied a bioinformatics approach to analyse the Cancer Genome Atlas (TCGA) and Gene Expression Omnibus (GEO) databases for differentially expressed lncRNAs in BC and normal paraneoplastic samples. We analysed *LINC00524* expression levels in BC samples and lines of BC cells and its effect on BC invasion, metastasis and proliferation in vitro and in vivo, while also conducting a preliminary exploration of its possible functions. Our findings indicate that *LINC00524* has been elevated in BC regions and highly invasive BC cells and corresponds to a poor prognosis for patients with BC. Furthermore, *LINC00524* can interact with TDP43 to promote BC invasion and metastasis, suggesting its potential as a novel clinical marker and molecular target against BC treatment.

## 2 MATERIALS AND METHODS

### 2.1 Collection of BC specimens and cell culture methodology

BC tissues and their adjacent normal counterparts were procured, post‐surgical procedures, from patients in Affiliated Hospital of Nantong University between January 2016 and December 2018, contingent on informed consent. Notably, these patients underwent no prior treatments like chemotherapy, radiotherapy or targeted immunotherapy. Ethical clearance for the research was acquired from the institution's ethics committee. BC cell lines, namely MCF‐7, MDA‐MB‐231, MDA‐MB‐468 and 4T1, were sourced from the Chinese Academy of Sciences, Shanghai. These cell lines were cultivated in specific media, supplemented with 10% FBS, 100 μg/mL penicillin and 100 μg/mL streptomycin, and consistently incubated at 37°C under 5% CO_2_.

### 2.2 Data acquisition and processing

The transcriptomic data of BC samples were retrieved from the TCGA (https://portal.gdc.cancer.gov) database and the GSE119233 data set from the GEO database (http://www.ncbi.nlm.nih.gov/geo/). LncRNA annotation followed the GRCh38 standards on the GENCODE platform (https://www.gencodegenes.org/). Perl (Strawberry Perl v5.28.1) is used for data processing. In total, 1096 clinical follow‐up records were extracted from the TCGA using UCSC Xena (https://xena.ucsc.edu/). Patients whose clinical information was unknown were deleted (*n* = 26), resulting in a final cohort of 1070 clinical BC samples for analysis. The limma package in R version 4.1 (R Foundation for Statistical Computing, Austria) was utilized to log2‐transform and normalize the data.^28^ Subsequently, the intersection of differentially expressed genes was determined using the Venn R package, and gene differential analysis was performed using the limma R package to compare BC and normal samples. Comprehensive data visualization was achieved using heatmaps and volcano plots, and survival trends were evaluated via Kaplan–Meier analyses. Furthermore, a Gene Ontology (GO) enrichment analysis was executed to discern lncRNA‐associated mRNA expression patterns using the Metascape portal (https://metascape.org/gp/index.html#/main/step1).

### 2.3 Plasmid construction and transfection

*LINC00524* interference plasmid (sh‐*LINC00524*), overexpression plasmid (pcDNA‐*LINC00524*) and a control vector (Table S1) were transfected into BC cells using Lipofectamine 2000 reagent (Invitrogen, USA), all of which were purchased from OBiO Technology (Shanghai, China).

### 2.4 Quantitative real‐time PCR (qRT‐PCR) analysis

RNA extraction from cultured cells was achieved via the TRIzol methodology (Invitrogen, USA). The reverse transcription of RNA to cDNA utilized the HiScript II RT SuperMix, followed by qPCR analyses employing Universal SYBR qPCR Master Mix (Vazyme, China). GAPDH served as the internal reference. Primer sequences are delineated in Table S2.

### 2.5 Western blot

BC cell protein extracts were processed with RIPA buffer (Beyotime Biotechnology, China) containing protease inhibitors (Meilunbio, China). Primary antibodies were obtained from Abcam (1:1000, TDP43, ab109535, USA) and Sigma‐Aldrich (1:10000, β‐actin, A5316, USA). The membranes were subsequently incubated with appropriate secondary antibodies, and an ECL detection system (Tanon, China) was employed to detect protein bands.

### 2.6 Cell migration and invasion analysis

Cell migratory capacity was determined through a wound healing assay as per established laboratory protocols.^29^ Forty‐eight hours post‐transfection, cells grew and converged to form a monolayer at the bottom of a 6‐well plate (Corning, USA). The monolayer was scratched with a sterile 200 μL pipette tip (KIRGRN, China) to create a wound. Cells were carefully washed with PBS and then replaced with serum‐free medium. After 24 h of incubation in a 37°C incubator containing 5% CO_2_, the wound area was measured again. Invasion capacities of cells were assessed using a transwell assay. BC cells at a specific density (5 × 10^4^ cells for MAD‐MB‐231 and 1 × 10^5^ cells for MCF‐7) were inoculated above an 8 μm transwell migration chamber (Corning, 3422, USA) coated with Matrigel (Biocoat, USA) in medium with 1% FBS, and below the chamber with 600 μL medium containing 10% FBS for 24 h. Invaded cells underwent fixation, staining and subsequent microscopic examination (Leica, Germany).

### 2.7 Cell proliferation assay

The capability of cells to proliferate was ascertained utilizing the colony formation assay coupled with the Cell Counting Kit‐8 (CCK‐8, Beyotime Biotechnology, China) assay. Post 48‐h transfection, cells of varying groups were plated in 6‐well plates at densities ranging from 5 × 10^2^ to 1 × 10^3^ cells per well under conditions of 5% CO_2_ at 37°C for a duration of 14 days to aid colony development. Colonies underwent fixation and subsequent staining with crystal violet. Quantification of colonies ensued to deduce proliferative potential. In accordance, 1000–2000 transfected BC cells/100 μL were seeded in 96‐well plates (Corning, USA). Sequential measurements were made post 0, 24, 48 and 72 h post the addition of 10 μL of CCK‐8 reagent and an hour of incubation. Absorbance values were procured at 450 nm using a microplate reader (Bio‐Tek, USA). Wells devoid of cells were designated as controls.

### 2.8 Tumour xenograft assay

BALB/c female mice aged 8 weeks, with a weight range of 22–25 g, were procured from the Experimental Animal Center of Nantong University (Institutional licence: SYXK(SU)‐2012‐0030). All murine experiments received approval from the Animal Ethics Committee of Nantong University (Approval No. S20201115‐902) and were in line with guidelines prescribed by the National Institutes of Health regarding the care and utilization of laboratory animals.^30^ 4T1 cells along with transfected pcDNA‐LINC00524 and corresponding control cells were grafted into the mammary fat pad of the mice (*n* = 6/group). Periodic measurements of tumour dimensions were made using a calliper, with volume calculations derived from the equation: π/6 × L × W^2^.^31^ Upon tumours achieving a size bracket of 1000–1500 mm^3^, mice underwent anaesthesia via intraperitoneal administration of sodium pentobarbital (Cat# P‐010, Millipore Sigma, USA) prior to tumour excision.

### 2.9 Immunohistochemistry and haematoxylin and eosin staining

Tissues derived from tumours and lungs were promptly fixed in 4% paraformaldehyde for a 24‐h span prior to paraffin embedding. Following the deparaffinization and hydration of these sections, antigen retrieval was executed utilizing sodium citrate. Subsequent steps involved incubation with 0.3% hydrogen peroxide and primary antibodies against TDP43 (1:100, ab109535, Abcam). Detection involved the use of DAB staining kit (DS‐0005, Zhongshan Jinqiao, China) and haematoxylin. For the imaging process, a DM4000B microscope (Leica, Germany) was employed.

### 2.10 RNA pull‐down assay and RNA immunoprecipitation (RIP)

Pull‐down assays were carried out with biotinylated *LINC00524* using a Pierce™ Magnetic RNA‐Protein Pull‐Down Kit (Thermo Fisher, USA). *LINC00524* and *LINC00524* antisense sequences (Table S3) were in vitro transcribed with the TranscriptAid T7 High Yield Transcription Kit (Thermo Fisher, USA) containing Biotin‐16‐UTP (Roche, USA). Biotin‐labelled RNAs were combined with MAD‐MB‐231 cell extract (containing 3 mg total protein) in 500 μL RIP Lysis buffer and incubated at 37°C for 1 h. Then, 50 μL of washed streptavidin‐magnetic beads were added to each reaction. After further incubating, the beads were briefly washed five times with 1 mL RIP Lysis buffer. The pull‐down complexes were eluted by denaturation in 2× protein loading buffer for 10 min at 95°C. The samples were detected by western blot or mass spectrometry identification. For the RIP process, cell lysates from MDA‐MB‐231 were incubated with 2 μg anti‐TDP43 antibody (Abcam) or 2 μg anti‐IgG (Beyotime Biotechnology), and the isolated RNA underwent qRT‐PCR analysis. Primers were listed in Table S4.

### 2.11 Statistical analysis

Both GraphPad Prism v8.0 and the R programming language (version 4.1) were the platforms for data analysis. Values are presented as mean ± standard deviation (SD). The chosen analytical strategies were dependent on data adherence to normal distribution and variance homogeneity. Bivariate comparisons were performed via the two‐sample *t*‐test or Mann–Whitney *U*‐test. Survival rates were computed using the Kaplan–Meier approach. Pearson's correlation was utilized to elucidate the relationship between LINC00524 and TDP43 protein expressions. A *p*‐value threshold of <0.05 was taken as the criterion for statistical significance. All experiments were replicated a minimum of three times.

## 3 RESULTS

### 3.1 Differential expression of lncRNAs in BC

**FIGURE 1:**
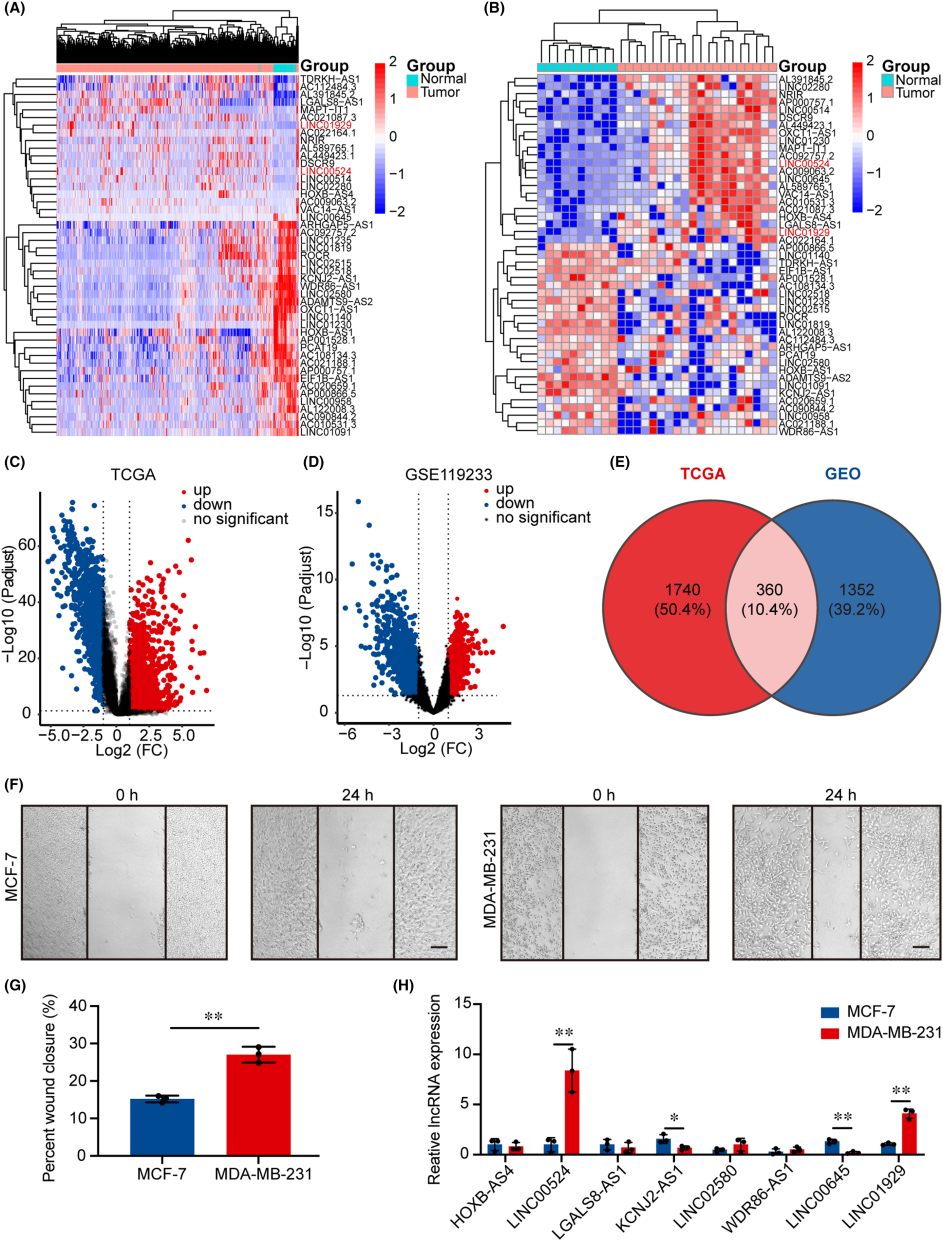
Differential expression of BC‐related lncRNAs. (A, B) Heatmap illustrating the expression of 47 lncRNAs with significant variation in BC tissues and adjacent normal tissues in the TCGA and GEO databases. A redder colour represents higher lncRNA expression, while a bluer colour signifies lower lncRNA expression. (C, D) Volcano plots depicting differentially expressed lncRNAs associated with BC in the TCGA and GEO databases (GSE119233). Red dots indicate upregulated genes, and blue dots represent downregulated genes. (E) In total, 360 lncRNAs with significantly differential expression were identified between the TCGA and GSE119233 data sets. (F) Images illustrating the wound‐healing capacity of MCF‐7 and MDA‐MB‐231 cells after 24 h. Scale bar = 200 μm. (G) Quantitative assessment of the wound closure efficiency of MCF‐7 and MDA‐MB‐231 cells (n=3). (H) qRT‐PCR‐determined statistical plots of the relative variations in lncRNA expression levels in MCF‐7 and MDA‐MB‐231 cells with variable migration abilities. **p < 0.01, *p < 0.05.

Following the extraction of 2100 lncRNAs with significant differential expression data from TCGA database, which contained samples from BC patients, 1075 lncRNAs were upregulated and 1025 lncRNAs were downregulated (|Log_2_FC| > 1, *p* < 0.05). Heatmap and volcano plots were employed to display genes correlated with BC tissue (*n* = 1096) and normal breast tissue (*n* = 112) that exhibited differential expression (Figure 1A,C). In the GEO database (GSE119233), which included 20 BC tissues and 10 normal breast tissues, 1712 lncRNAs with significant differences were identified. Differentially expressed lncRNAs are represented by a heatmap (Figure 1B) and a volcano plot (Figure 1D). A total of 360 significantly different lncRNAs were identified in BC and adjacent normal tissues across the two databases (Figure 1E; Figure S1A; Table S5). The human BC cell lines MDA‐MB‐231 and MCF‐7 exhibited differences in aggressiveness and malignancy, with MDA‐MB‐231 being highly metastatic and malignant.^29^, ^32^ This finding was also corroborated by a wound healing assay (Figure 1F,G). From the 360 differentially expressed lncRNAs, we selected 8 lncRNAs with the largest fold change for qRT‐PCR analysis and KM analysis (Figure S1B–G; Table S2). Analysis of cell lines with varying migration abilities demonstrated that lncRNA *LINC00524* and *LINC01929* expression were substantially increased in MDA‐MB‐231 cells, while *lncRNA KCNJ2‐AS1* and *LINC00645* were markedly downregulated in MCF‐7 cells (Figure 1H). Collectively, the specific higher expression level of *LINC00524* in cell lines with increased migration capacity prompted us to investigate whether *LINC00524* is critical in BC.

### 3.2 *LINC00524* upregulation in BC and association with cell migration via lncRNA–protein interactions

**FIGURE 2:**
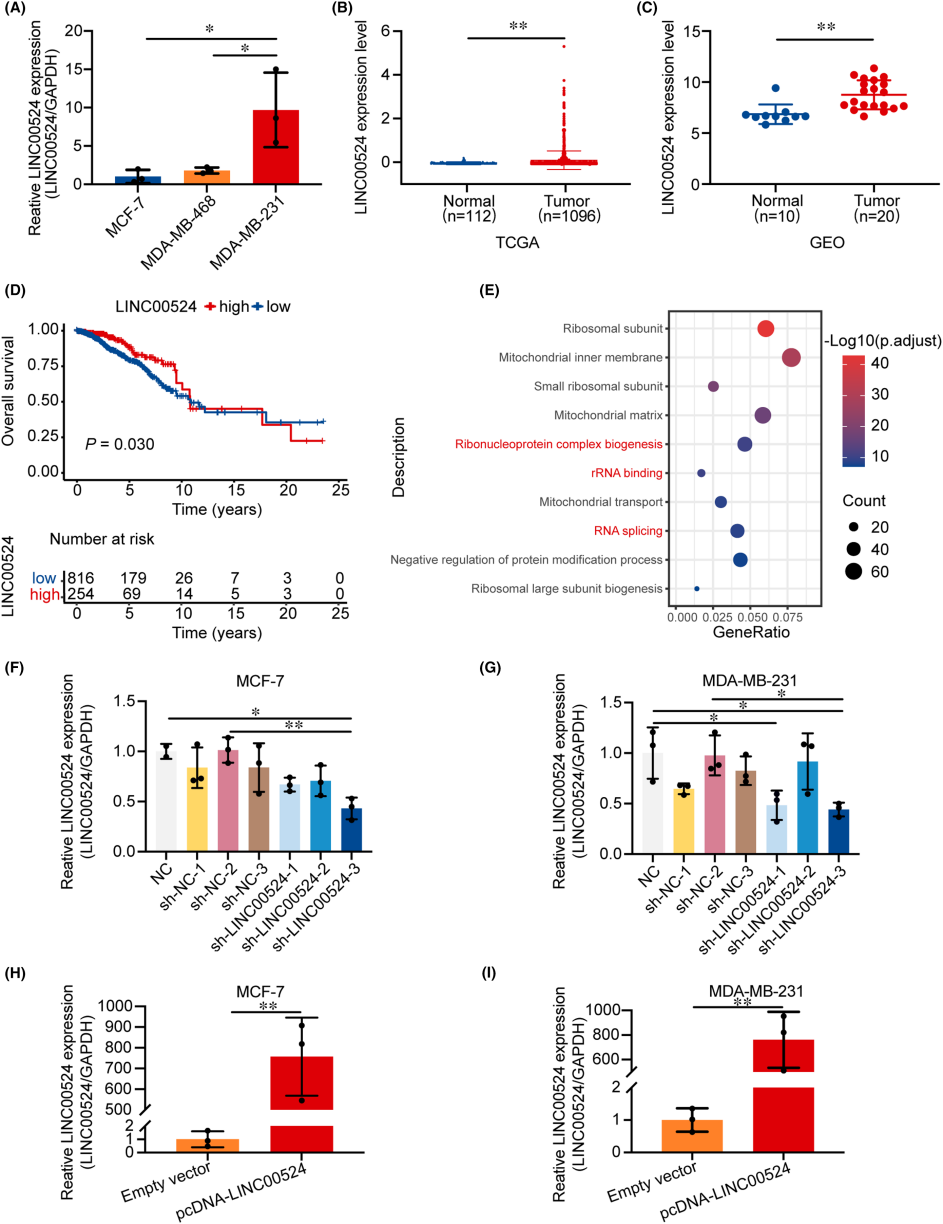
Expression of *LINC00524* in BC and its prognostic significance in BC patients. (A) *LINC00524* expression in BC cell lines (MCF‐7, MDA‐MB‐468 and MDA‐MB‐231) detected by qRT‐PCR. (B, C) *LINC00524* expression in the TCGA and GSE119233 cohorts. (D) In the TCGA data, a high level of *LINC00524* expression was associated with a poorer overall survival time, as determined by a Kaplan–Meier analysis of survival (*n* = 1070). (E) GO analysis was performed on the *LINC00524*‐associated genes from the TCGA data set (*n* = 1025). (F, G) The expression levels of *LINC00524* in MCF‐7 and MDA‐MB‐231 cells following transfection with sh‐NC or sh‐*LINC00524* were detected by RT‐PCR, where NC is a negative control. (H, I) qRT‐PCR was used to determine the ectopic expression of *LINC00524* in *LINC00524*‐transfected MCF‐7 and MDA‐MB‐231 cells. NC, control group; Empty vector, *LINC00524* empty group; pcDNA‐*LINC00524*, *LINC00524* overexpression group; sh‐NC, *LINC00524* interference empty group; sh‐*LINC00524*, *LINC00524* interference group. **p < 0.01, *p < 0.05.

Subsequently, we explored the expression level of *LINC00524* in BC (Figure S2A). The qRT‐PCR analysis of three cell lines revealed that the expression of *LINC00524* in BC cells with high migration capacity (MDA‐MB‐231) was significantly higher than that in cells with low migration capacity (MCF‐7) (Figure 2A). Therefore, we predicted that *LINC00524* could be essential in BC migration and selected low‐ and high‐expressing MCF‐7 and MDA‐MB‐231 cells, respectively, for further functional studies. *LINC00524* was substantially elevated in BC samples, and the differential levels of *LINC00524* were the most notable in both TCGA and GEO databases (GSE119233) (Figure 2B,C). Additionally, BC patients with high *LINC00524* expression exhibited poor prognosis (Figure 2D). Following GO enrichment analysis, *LINC00524* was found to be predominantly involved in ribonucleoprotein complex biogenesis, rRNA binding and RNA splicing (Figure 2E). To find out the biological function of *LINC00524* in BC, we designed three different *LINC00524* interfering short hairpin RNA (shRNA) plasmids and an overexpression plasmid for transfection into MCF‐7 and MDA‐MB‐231 cell lines (Table S1). The results showed that sh‐*LINC00524*‐3 exhibited the best efficiency in both BC cell lines (Figure 2F,G). Moreover, we constructed a *LINC00524* overexpression vector using pcDNA3.1 and observed that expression level of *LINC00524* was substantially enhanced in BC cells following transfection (Figure 2H,I). These results demonstrated that *LINC00524* has increased expression in BC tissues and is connected with a poor prognosis for BC patients, potentially influencing BC progression by promoting migration through lncRNA–protein interactions.

### 3.3 *LINC00524* association with BC cell migration and invasion

**FIGURE 3:**
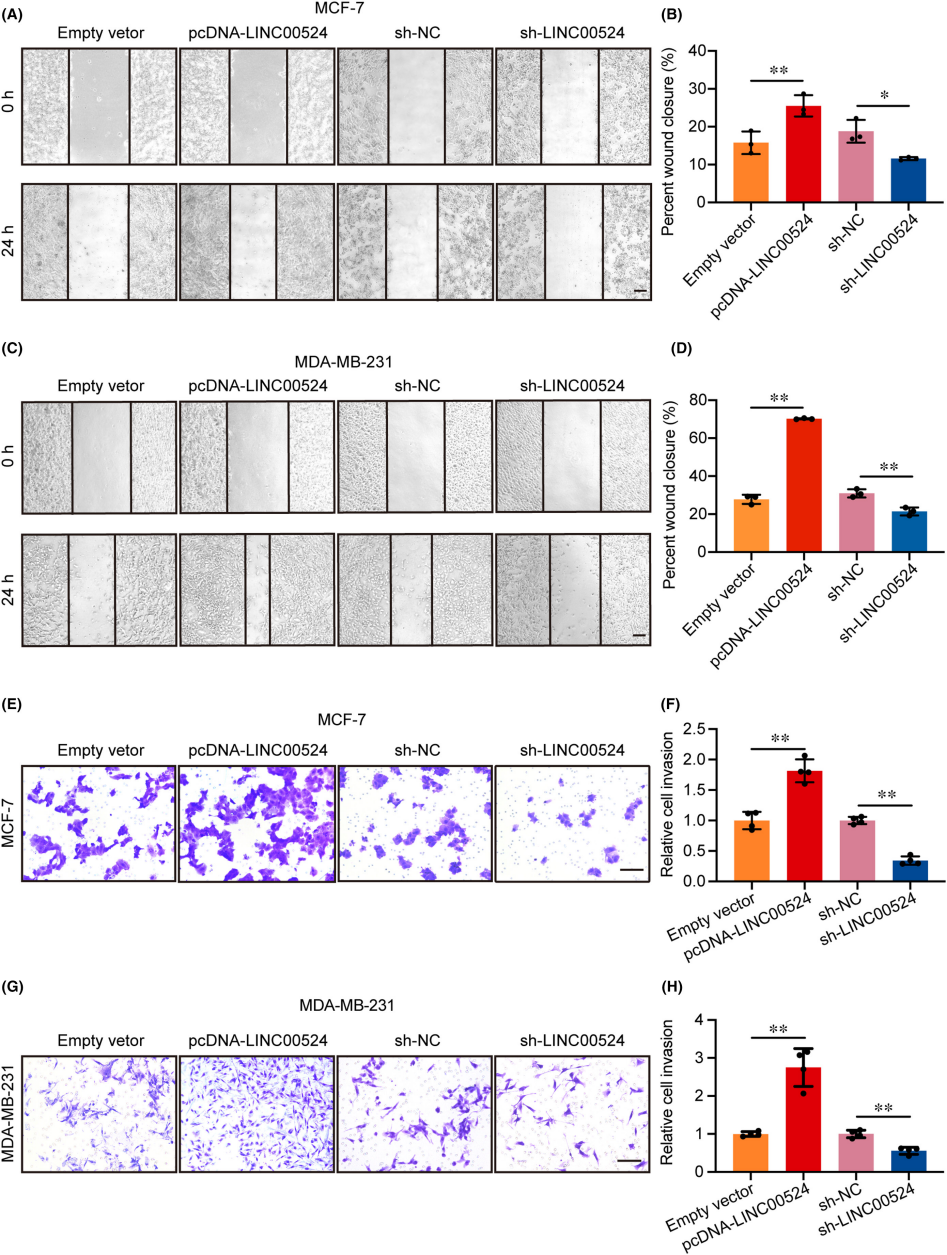
Effect of *LINC00524* on BC cell migration and invasion in vitro. (A, B) The wound‐healing assay was used to evaluate the migration capacity of transfected BC cell lines with *LINC00524* overexpression and *LINC00524* interference, as well as empty vector‐transfected cells. Scale bar = 200 μm. (C, D) Quantitative assessment of the wound closure efficiency (*n* = 3). (E, F) Transwell assays were utilized to evaluate the invasion ability of MCF‐7 and MDA‐MB‐231 cells with *LINC00524* overexpression, *LINC00524* interference and empty vector transfection. Scale bar = 100 μm. (G, H) Statistical analysis of the invasion ability of cells (*n* = 3). Empty vector, *LINC00524* empty group; pcDNA‐*LINC00524*, *LINC00524* overexpression group; sh‐NC, *LINC00524* interference empty group; sh‐*LINC00524*, *LINC00524* interference group. *p < 0.05; **p < 0.01.

Previous research predicted that *LINC00524* may be involved in the migration of BC cells. In pursuit of discerning the function of *LINC00524* within BC, a systematic experimental approach was employed. When *LINC00524* was overexpressed, the ability of BC cells to migrate significantly increased. However, the wound healing assay revealed that the migration capacity of cells transfected with sh‐*LINC00524* was significantly diminished (Figure 3A–D). The transwell assay also revealed increased cell invasion capacity adhering to *LINC00524* overexpression, whereas reduction of *LINC00524* expression decreased MCF‐7 and MDA‐MB‐231 cells invasion capacities (Figure 3E–H).

### 3.4 *LINC00524* association with BC cell proliferation

**FIGURE 4:**
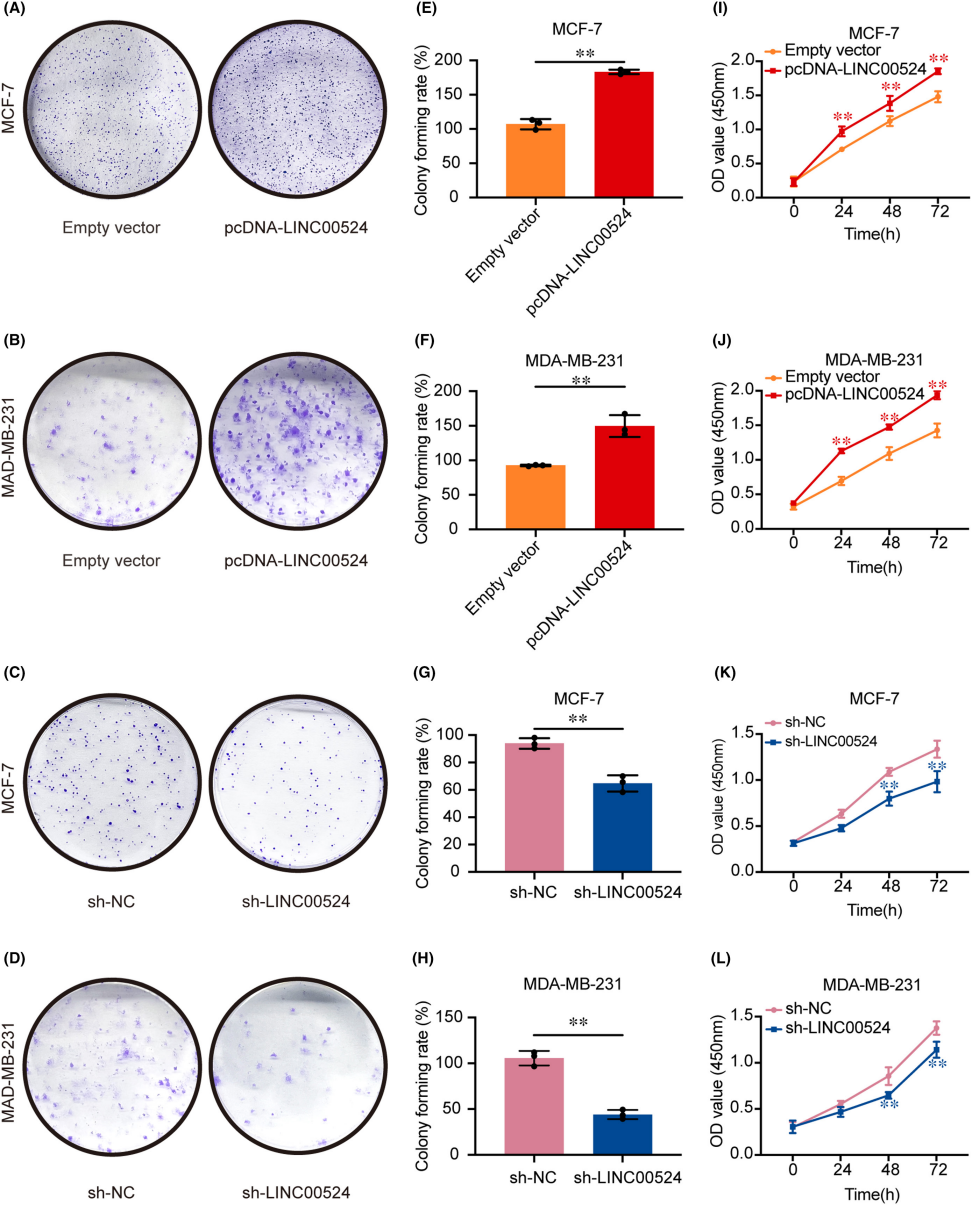
Effect of *LINC00524* on BC cell proliferation in vitro. (A–D) The colony formation assay was harnessed to gauge the proliferative aptitude of cells post‐transfection with *LINC00524* overexpression, *LINC00524* silencing and empty vector. (E–H) Statistical analysis of the colony formation potential of MCF‐7 and MDA‐MB‐231 cells (*n* = 3–4). (I–L) CCK‐8 assay in pcDNA‐*LINC00524* or sh‐*LINC00524* transfected MCF‐7 and MDA‐MB‐231 cells (*n* = 4). Empty vector, *LINC00524* empty group; pcDNA‐*LINC00524*, *LINC00524* overexpression group; sh‐NC, *LINC00524* interference empty group; sh‐*LINC00524*, *LINC00524* interference group. **p < 0.01.

To delve deeper into the biological significance of *LINC00524*, proliferation assays were systematically carried out. Employing both the colony formation and CCK‐8 assays, variations in cellular proliferative tendencies were charted. BC cells, when augmented with *LINC00524* expression, manifested a heightened propensity for colony establishment as opposed to those transfected with an empty vector. Conversely, suppression of *LINC00524* was associated with a diminishment in colony number and dimensions, particularly evident in the MCF‐7 and MDA‐MB‐231 BC cell lines when juxtaposed against the empty vector‐transfected cohorts (Figure 4A–H). The CCK‐8 assay further underscored an elevated proliferation rate in BC cells upon pcDNA‐*LINC00524* transfection, whereas *LINC00524* interference was concomitant with a substantial decrement in proliferative capacity in the aforementioned cell lines (Figure 4I–L).

### 3.5 *LINC00524* regulates BC through interaction with TDP43

**FIGURE 5:**
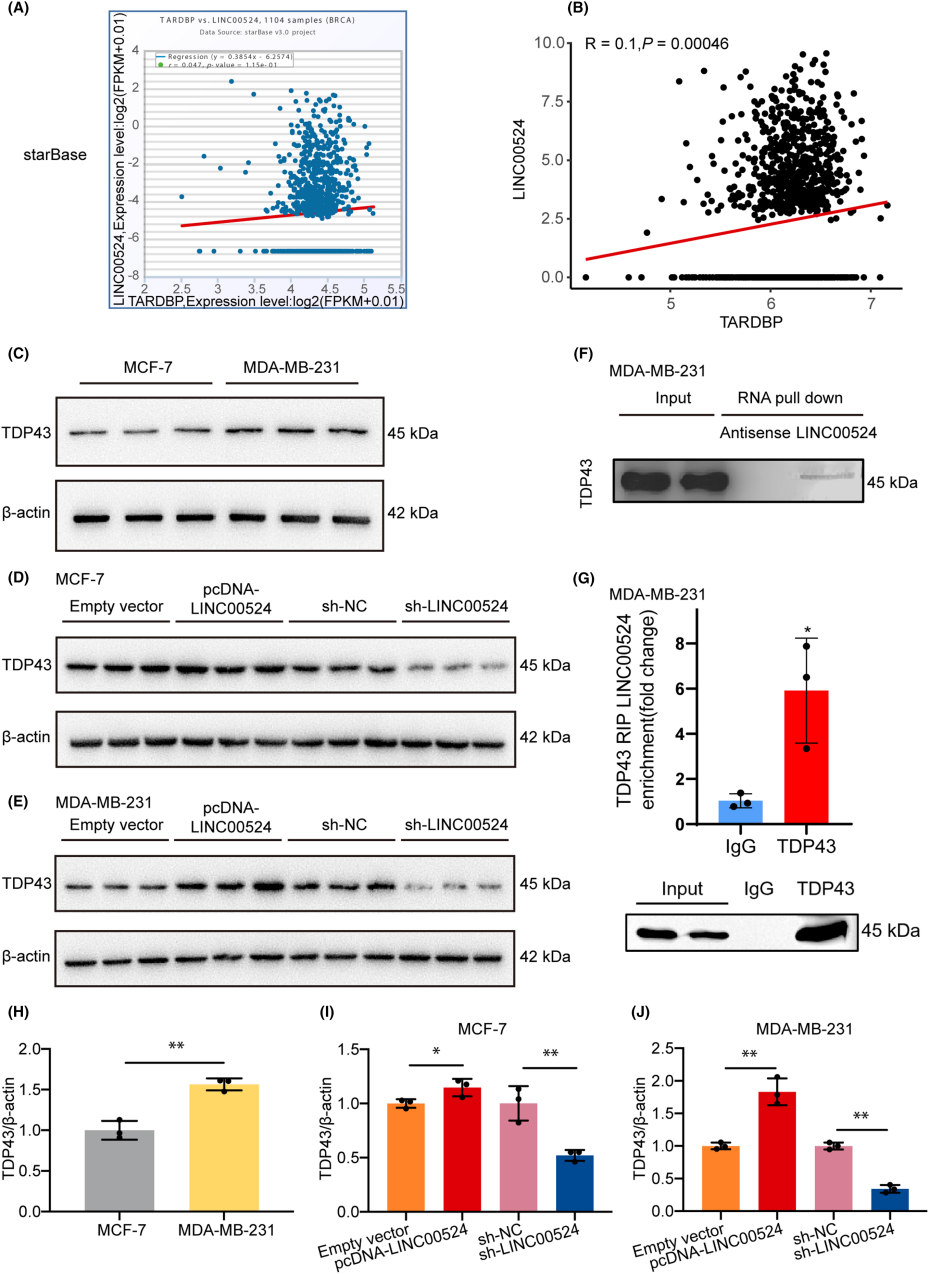
*LINC00524* Binds to the RNA‐binding protein TDP43. (A) Correlation analysis of *LINC00524* and TDP43 in the Starbase BC database (*n* = 1104). (B) Correlation analysis of *LINC00524* and TDP43 in the TCGA database (*n* = 1208). (C) Western blot analysis of *LINC00524* expression in MCF‐7 and MDA‐MB‐231 BC cell lines. (D, E) Western blot investigations determined both the overexpression and silencing of *LINC00524* across the transfected entities and the consequent expression of TDP43 in empty vector‐transfected MCF‐7 and MDA‐MDA‐231 cells. (F) Detailed western blot analysis of the intricate relationship between TDP43 and biotin‐tagged *LINC00524* using three distinct streptavidin RNA pull‐down assays. (G) Western blot results of RIP products showed that the experiment using TDP43 as bait protein to obtain TDP43‐*LINC00524* complex was successful. (H) Statistical analysis of TDP‐43 expression in MCF‐7 and MDA‐MB‐231 BC cell lines (*n* = 3). (I, J) Statistical analysis of TDP43 expression in the overexpression and silencing of *LINC00524* transfected lines and the expression of TDP43 in empty vector‐transfected MCF‐7 and MDA‐MB‐231 cells (*n* = 3). Empty vector, *LINC00524* empty group; pcDNA‐*LINC00524*, *LINC00524* overexpression group; sh‐NC, *LINC00524* interference empty group; sh‐*LINC00524*, *LINC00524* interference group. **p < 0.01, *p < 0.05.

To further investigate the specific mechanism by which *LINC00524* promotes BC invasion and metastasis, we first predicted proteins that may interact with *LINC00524* using the starBase (ENCORI) online database (https://starbase.sysu.edu.cn/index.php). Based on the database BC correlation analysis (*n* = 1104), the protein target with the most significant correlation coefficient, TDP43 (TARDBP), was selected for subsequent prediction analysis (Figure 5A). A correlation analysis based on the TCGA database of BC patients (*n* = 1208) revealed a significant positive correlation between *LINC00524* and TDP43 expression (Figure 5B). Using western blot analysis, we detected the expression of TDP43 in MCF‐7 and MDA‐MB‐231 cells and found that the relative protein expression of TDP43 was substantially higher in the MDA‐MB‐231 group than in the MCF‐7 group (Figure 5C,H), consistent with our detection of *LINC00524* exhibiting a similar expression trend in both cells. Upon *LINC00524* overexpression, its expression was enhanced, while interference led to a notable suppression in both MCF‐7 and MDA‐MB‐231 cells (Figure 5D,E,I,J). These results suggest that *LINC00524* regulates the expression of TDP43 in BC cells. To identify the protein partners binding to *LINC00524*, we performed RNA pull‐down assays. MDA‐MB‐231 cell lysates were incubated with biotinylated, in vitro‐transcribed *LINC00524* or its antisense RNA. TDP43 was identified by mass spectrometry as a significant protein in *LINC00524* pull‐down samples under both conditions but not in antisense samples (Figure 5F). This was further substantiated by RIP assays where *LINC00524* displayed significant enrichment by the TDP43 antibody (Figure 5G).

### 3.6 *LINC00524* 's involvement in BC invasion and metastasis via TDP43 interaction in vivo and prognostic implications of TDP43 expression

**FIGURE 6:**
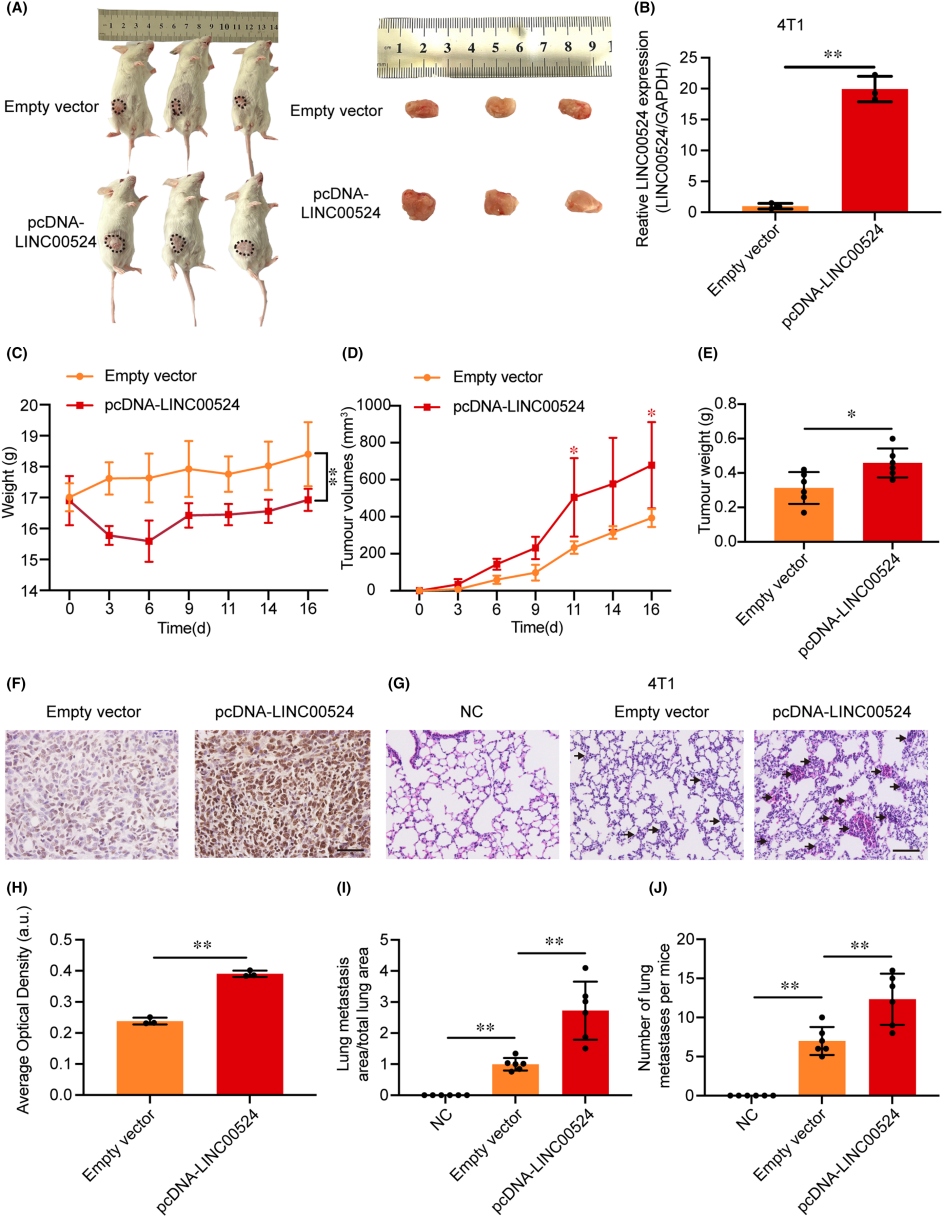
*LINC00524* interacts with TDP43 in vivo to promote BC metastasis. (A) 4T1 cells, post‐transfection with either the *LINC00524* overexpression vector or its control counterpart, were administered into murine mammary adipose tissues. (B) qRT‐PCR analysis discerned *LINC00524* expression in 4T1 entities post‐transfection with either the empty vector or pcDNA‐*LINC00524* (*n* = 3). (C) Overexpression of *LINC00524* in mice resulted in significant weight loss (*n* = 6). (D, E) Augmentation in both tumour magnitude and mass was observed in the *LINC00524* overexpression cohort (*n* = 6). (F) Representative images of immunohistochemical analysis of TDP43 expression in the xenograft tumour. Scale bar = 50 μm. (G) Illustrative captures of haematoxylin and eosin staining of murine pulmonary tissues. (H) Statistical plots of the analysis of the average optical density values of TDP43 expression in the overexpressed *LINC00524* group and empty vector (*n* = 3). (I, J) Number of pulmonary metastatic nodules and proportion of lung metastasis area relative to the total lung area in each group (*n* = 6). NC, control group; Empty vector, *LINC00524* empty group; pcDNA‐*LINC00524*, *LINC00524* overexpression group. **p < 0.01, *p < 0.05.

To further study the role of *LINC00524* in vivo, We established an orthotopic breast metastasis model by injecting 4T1 cells expressing *LINC00524* or a control vector into the adipose breast pads of mice (Figure 6A, Figure S2B). qRT‐PCR demonstrated a significant increase in *LINC00524* expression in the 4T1 cells in the overexpression *LINC00524* group contrasting to the empty vector group (Figure 6B). We monitored the mice's body weight and tumour size, and the results showed that mice overexpressing *LINC00524* had significantly reduced body weight relative to the control group (Figure 6C). The tumour growth rate and tumour weight of the *LINC00524* overexpression group were significantly higher than the empty vector groups (Figure 6D,E). After tumour separation, immunohistochemical staining revealed that TDP43 expression increased in tumours overexpressing *LINC00524* (Figure 6F,H). To further illustrate that *LINC00524* can affect BC metastasis in vivo, we selected lung tissue as the study tissue for its ease of observation. In the *LINC00524* overexpression group, the number and area of pulmonary metastatic nodules were more significant than in the other two groups (negative control and empty vector) (Figure 6G,I,J). To further investigate the connection involving TDP43 expression and clinical BC, we employed tissue microarray technology to observe the expression of TDP43 in BC patients with different pathological features (Figure S3A–C), and according to the results, 51% of BC tissues were positive for TDP43 expression (29/57). At the Affiliated Hospital of Nantong University, we collected prognostic data from 125 BC patients (66 patients with high TDP43 expression and 59 patients with low TDP43 expression). Among the 125 BC patients (Table 1), TDP43 expression corresponded significantly with tumour size (*χ* ^2^ = 7.586, *p* = 0.006), ER (oestrogen receptor) (*χ* ^2^ = 15.879, *p* = 0.0001) and Ki67 (*χ* ^2^ = 16.019, *p* = 0.0001). Nonetheless, no significant correlation existed between TDP43 and age, tumour grading, PR (progesterone receptor), HER2 (human epidermal growth factor receptor 2), lymphatic metastasis or TNM. In summary, *LINC00524* interacts with TDP43 to promote BC metastasis, and TDP43 expression portends an unfavourable clinical prognosis in BC.

**TABLE 1:** Relations between TDP43 and clinicopathological parameters of BC.

Features	Total	TDP43 expression		*χ* ^2^	*p* Value
		Low expression	High expression		
Age (years)					
≤40	11	4	7	4.424	0.109
40 ~ 60	75	39	36		
≥ 60	39	16	23		
Grading of tumours					
I ~ II	104	53	51	2.175	0.14
III	21	7	14		
Tumour size (cm^3^)					
≤ 2	60	36	24	7.586	0.006
> 2	65	23	42		
ER					
Negative	55	37	18	15.879	0.0001
Positive	70	22	48		
PR					
Negative	85	41	44	0.114	0.735
Positive	40	18	22		
HER2					
Negative	99	46	53	0.103	0.748
Positive	26	13	13		
Ki67					
Low	59	39	20	16.019	0.0001
High	66	20	46		
Lymphatic metastasis					
N0	85	44	41	2.221	0.136
N1 + 2 + 3	40	15	25		
TNM					
I	36	19	17	0.773	0.679
II	65	30	35		
III	24	10	14		

## 4 DISCUSSION

BC is a leading cause of cancer‐related mortality and presents an important health risk for women.^2^ The metastasis of BC continues to be a primary obstacle in its treatment, and the mechanisms underlying this process remain poorly understood.^33^ Increasing evidence, based on bioinformatics analysis, suggests that long non‐coding RNAs (lncRNAs) have been connected with malignant development and may serve as tumour biomarkers.^34^, ^35^, ^36^, ^37^, ^38^ This research analysed TCGA and GEO databases using bioinformatics techniques to identify lncRNAs with differential expression in BC and normal tissues. Our findings revealed that *LINC00524* is upregulated in BC and highly expressed in the migratory cells. Furthermore, high *LINC01929* expression correlates with lower survival rates among BC patients. GO enrichment analysis and these results suggest that *LINC00524* may contribute to BC migration by interacting with proteins, thereby influencing disease progression.

In the context of cancer, lncRNAs participate in diverse physiological and pathological processes.^39^, ^40^, ^41^ These molecules exhibit cancer‐specific expression patterns and participate in the development and invasive metastasis of several malignancies.^40^, ^42^, ^43^ Their high expression can both promote and inhibit tumour metastasis.^36^, ^44^ Current biochemical analyses have demonstrated that *LINC00524* is overexpressed in oral squamous and renal clear cell carcinoma relative to adjacent normal tissues and links with a poor prognosis for the patient. However, further in vitro and in vivo experimental studies are required to validate these findings.^45^, ^46^ The functional roles and specific targets of *LINC00524* in BC, both in vitro and in vivo, remain to be elucidated. Numerous studies have revealed that aberrantly expressed lncRNAs differentially regulate BC cell proliferation, invasion and metastasis.^47^, ^48^ To investigate systematically the effect of *LINC00524* on BC cell function, we constructed overexpression and interference systems for this lncRNA. Our in vitro experiments demonstrated that *LINC00524* overexpression promotes BC cell migration, invasion and proliferation, while interference inhibits these processes. Similarly, in vivo experiments revealed that *LINC00524* significantly enhances BC tumour proliferation and lung metastasis and is stably expressed in mice, exhibiting species conservation.

We further investigated the mechanistic role of *LINC00524* in promoting BC proliferation and metastasis. A significant positive correlation between *LINC00524* and TDP43 expression was identified through bioinformatics analysis.^49^ Additionally, TDP43 exhibited elevated levels of expression in triple‐negative breast cancer (TNBC) with poor prognosis, which is consistent with the upregulated *LINC00524* expression in TNBC cell lines, such as MDA‐MB‐231.^50^ Furthermore, TDP43 overexpression promoted breast epithelial cell proliferation and malignancy, while TDP43 knockdown suppressed tumour progression, including proliferation and metastasis. These findings align with our prior observations of *LINC00524* function, prompting us to further examine the relationship between *LINC00524* and TDP43. Our results revealed a significant upregulation of TDP43 protein expression in MDA‐MB‐231 cells. We also found that alterations in *LINC00524* expression were accompanied by changes in TDP43 protein levels. The RNA pull‐down assay demonstrated an interaction between *LINC00524* and TDP43 protein, and in vivo immunohistochemical staining revealed increased TDP43 expression in tumour tissues overexpressing *LINC00524*, which promoted BC lung metastasis. These findings suggest that *LINC00524* interacts with TDP43 and positively regulates TDP43 expression to enhance BC metastasis. Utilizing breast cancer tissue microarray technology and histopathological samples from 125 clinical BC patients, we assessed TDP43 expression and its association with clinicopathological characteristics of BC via immunohistochemistry. We observed that elevated TDP43 expression correlated with tumour size, ER status, and Ki67 levels, further elucidating the clinical relevance of TDP43 in BC. Future research will focus on an in‐depth investigation of the *LINC00524*‐TDP43 axis.

This study is the first to report the role of the novel long non‐coding RNA *LINC00524* in BC invasion, metastasis and proliferation (Figure S4). Concurrently, TDP43 expression was discovered to be correlated with a poor prognosis in patients with BC, confirming that *LINC00524* and TDP43 interact to modulate BC metastasis through an RNA–protein interaction mechanism. These findings offer new possibilities for the clinical diagnosis and treatment of BC based on molecular biology.

## AUTHOR CONTRIBUTIONS

**Xianglin Sun:** Data curation (equal); investigation (equal); methodology (lead); writing – original draft (equal). **Wenfeng Li:** Data curation (equal); methodology (equal); visualization (equal). **Gang Li:** Investigation (equal); methodology (equal). **Huan Yang:** Investigation (equal); methodology (equal); project administration (equal). **Zhenglin Jiang:** Resources (equal); supervision (equal); validation (equal). **Lihua Shen:** Resources (equal); visualization (equal). **Yuexiang Shen:** Formal analysis (equal); software (equal); visualization (equal). **Yifei Liu:** Resources (equal); supervision (equal); validation (equal). **Guohua Wang:** Funding acquisition (lead); investigation (lead); project administration (lead); supervision (lead); writing – original draft (supporting); writing – review and editing (lead).

## FUNDING INFORMATION

This study was generously funded by the National Natural Science Foundation of China under grant number 82171190, the Natural Science Foundation of Jiangsu Province of China via grant number BE2018778 and the Nantong Science and Technology Project with grant reference MS22021010.

## CONFLICT OF INTEREST STATEMENT

The authors of this manuscript declare no potential conflicts of interest.

## CONSENT TO PARTICIPATE

Every participant in the study granted their informed consent in a written format.

## CONSENT FOR PUBLICATION

All authors know and agree with the content of the paper and are listed as co‐authors of the paper.

## Supporting information


Figure S1

Table S1

## Data Availability

All data generated or analysed during this study are included as supplementary files.
