# Supplementary material for: A novel long non‐coding RNA LINC00524 facilitates invasion and metastasis through interaction with TDP43 in breast cancer
Source: J Cell Mol Med. 2024 Apr 3;28(8):e18275. doi: 10.1111/jcmm.18275 (PMC10989564; doi:10.1111/jcmm.18275)
Supplement: Supplementary file 1 — Figure S1 [file JCMM-28-e18275-s001.zip › FiguresS1-S4.docx]

**Fig. S1.** Examination of differentially expressed lncRNAs in BC. (A) A schematic representation of the bioinformatics analytical procedure undertaken in this investigation. (B-F) Kaplan-Meier survival analyses illustrating that elevated expression levels of *HOXB-AS4*, *LGLS8-AS1*, *KCNJ2-AS1*, *LINC02580*, and *WDR86-AS1*, respectively, are each associated with diminished overall survival durations in the TCGA dataset (n = 1070 for each analysis). (G) Kaplan-Meier survival examination indicating that an increased expression of *LINC00645* correlates with unfavorable overall survival duration in the TCGA dataset (n = 1070).

**Fig. S2.** *LINC00524* is a conserved lncRNA. (A) The *LINC00524* resides on chromosome 14 in humans. (B) Graphical views showing multi‐species comparisons of *LINC00524* using UCSC genome browser.

**Fig. S3.** TDP43 expression was associated with poor prognosis in BC. (A) Immunohistochemical evaluation for the detection of *LINC00524* expression within BC tissue specimens (n = 57). (B) Representative images from two BC cases were shown. Scale bar = 50 μm. (C) Representative images from BC cases were shown. Scale bar = 50 μm.

**Fig. S4.** Conceptual diagram illustrating the functional interplay of *LINC00524*/TDP43 in BC. A schematic portrayal elucidating the role and interaction of *LINC00524* and TDP43 within the context of breast cancer, offering insight into their potential mechanistic contributions to disease pathology.
