# Supplementary figures and images for: A novel long non‐coding RNA LINC00524 facilitates invasion and metastasis through interaction with TDP43 in breast cancer
Source: J Cell Mol Med. 2024 Apr 3;28(8):e18275. doi: 10.1111/jcmm.18275 (PMC10989564; doi:10.1111/jcmm.18275)

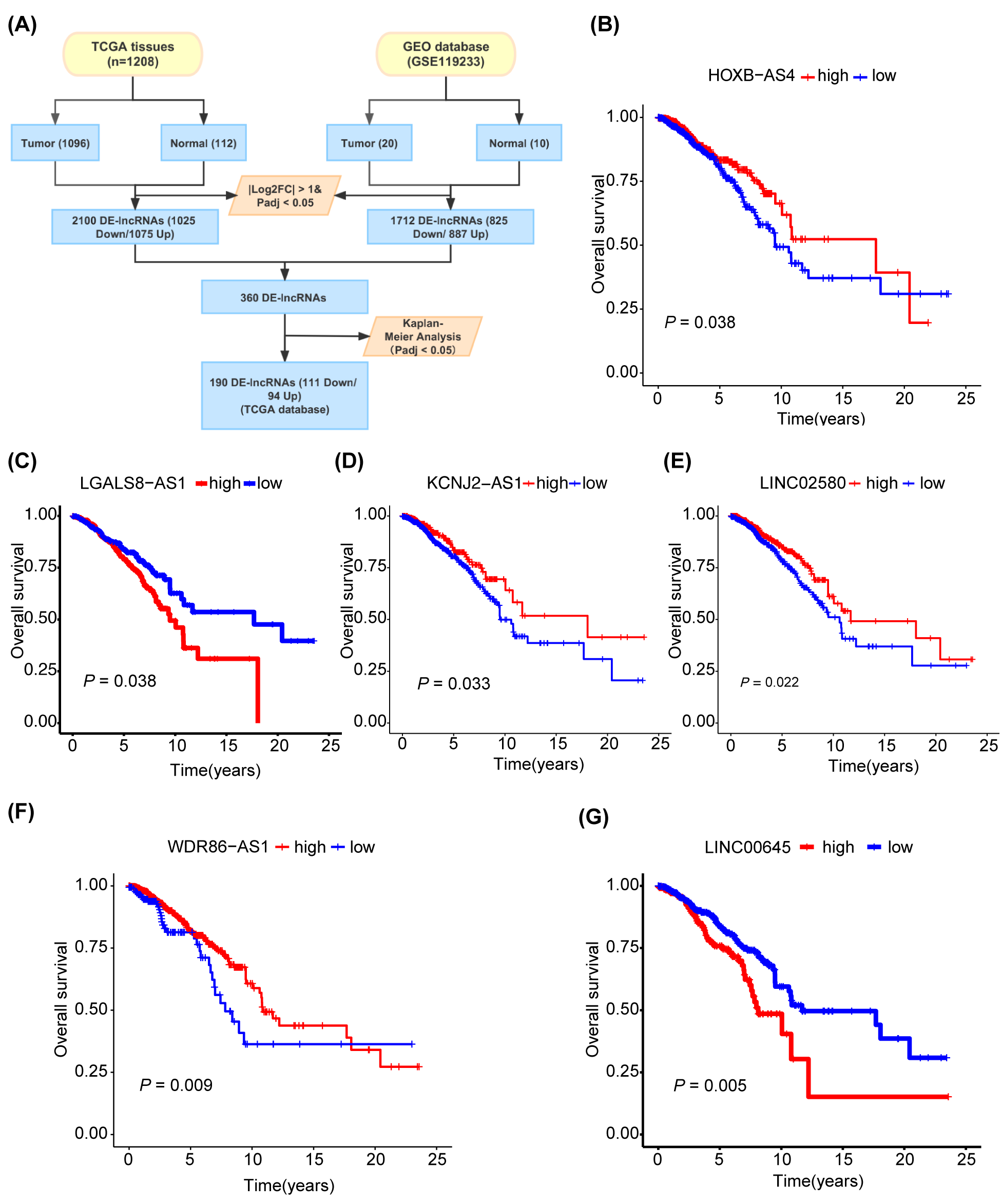

Supplement: Supplementary file 1 — Figure S1 [file JCMM-28-e18275-s001.zip › Figure S1.tif]

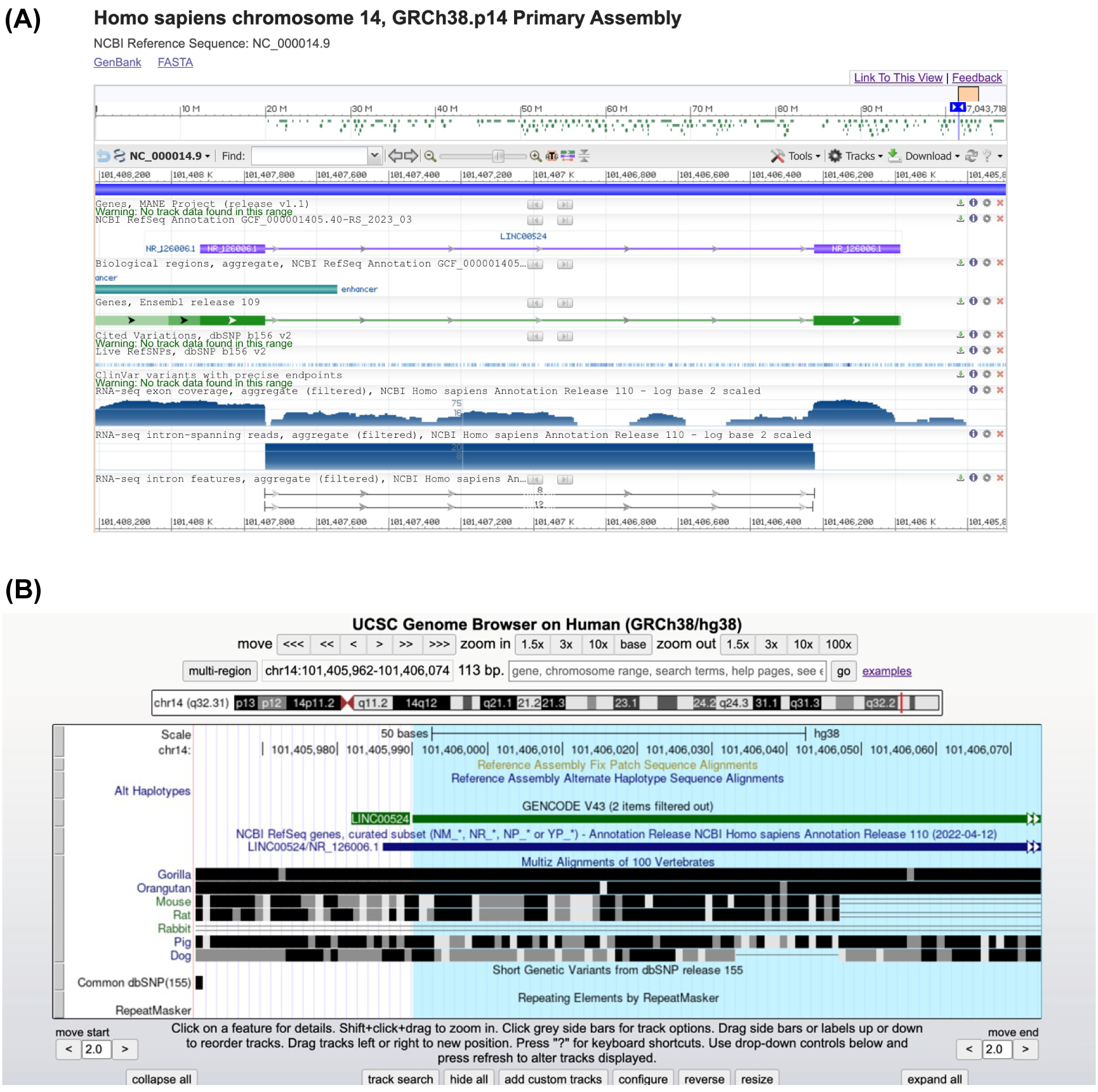

Supplement: Supplementary file 1 — Figure S1 [file JCMM-28-e18275-s001.zip › Figure S2.tif]

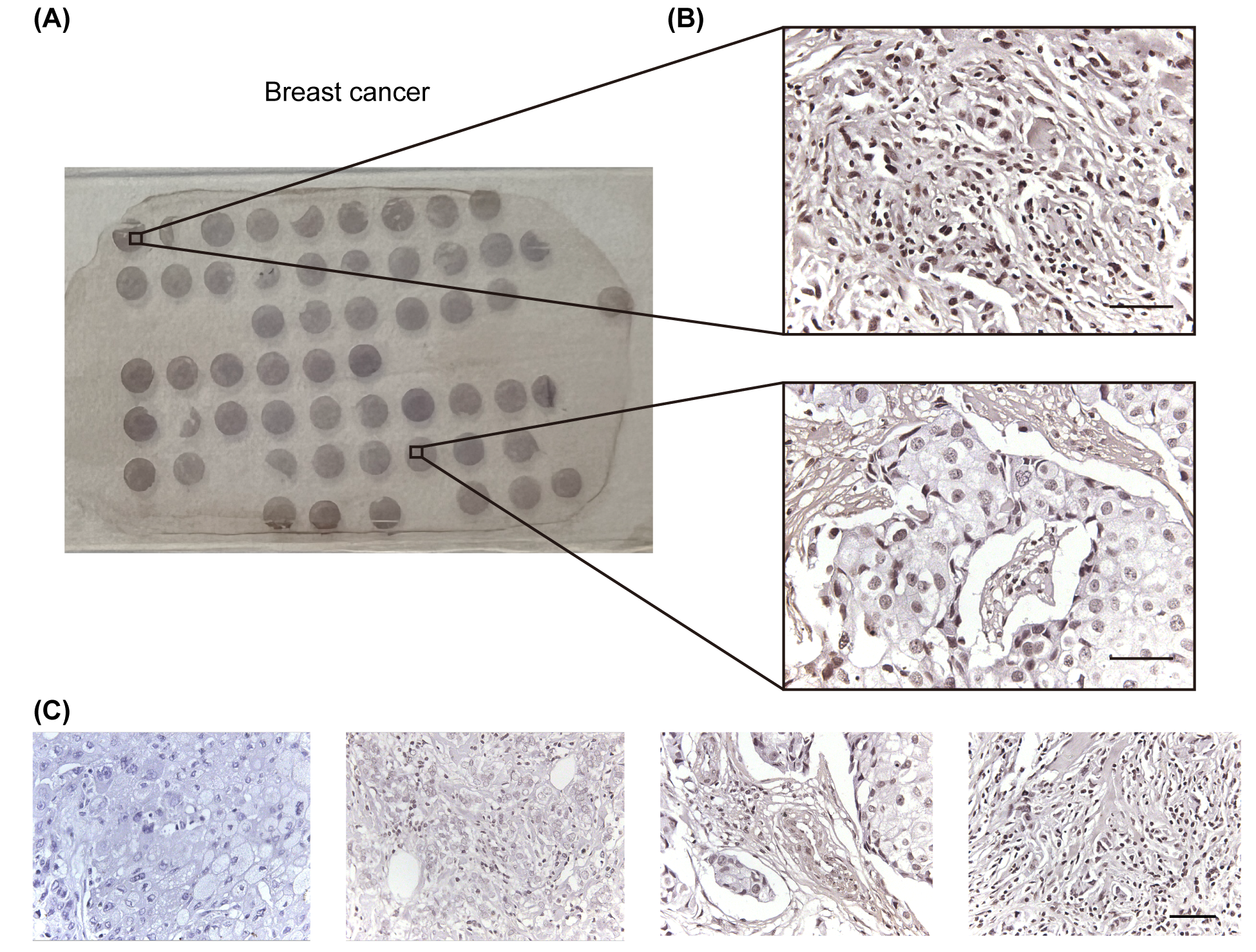

Supplement: Supplementary file 1 — Figure S1 [file JCMM-28-e18275-s001.zip › Figure S3.tif]

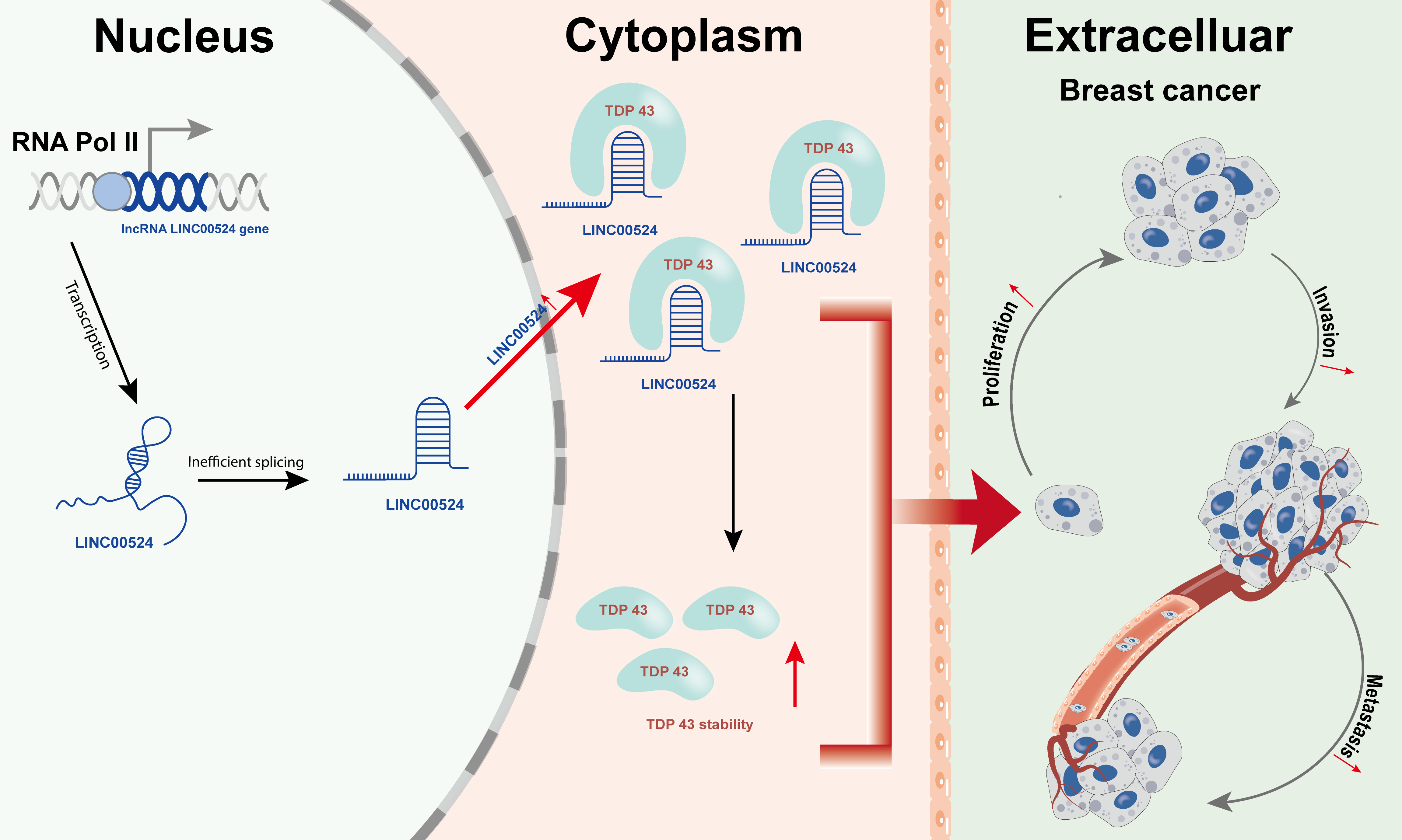

Supplement: Supplementary file 1 — Figure S1 [file JCMM-28-e18275-s001.zip › Figure S4.tif]
