# Supplementary material for: A novel long non‐coding RNA LINC00524 facilitates invasion and metastasis through interaction with TDP43 in breast cancer
Source: J Cell Mol Med. 2024 Apr 3;28(8):e18275. doi: 10.1111/jcmm.18275 (PMC10989564; doi:10.1111/jcmm.18275)
Supplement: Supplementary file 2 — Table S1 [file JCMM-28-e18275-s002.docx]

**Supplemental Tables**

**Table S1. Components used for plasmid construction**

| **Plasmid** | **Post-construction sequence components** |
| --- | --- |
| pcDNA-LINC00524 | pcDNA3.1（+）-LINC00524 |
| sh-LINC00524 | pSLenti-U6-shRNA (LINC00524)-CMV-EGFP-F2A-Puro-WPRE |
| Empty vector | pcDNA3.1（+） |
| sh-NC | pSLenti-U6-shRNA (NC2)-CMV-EGFP-F2A-Puro-WPRE |

Empty vector, LINC00524 empty group; pcDNA-LINC00524, LINC00524 overexpression group; sh-NC, LINC00524 interference empty group, sh-LINC00524, LINC00524 interference group.

**Table S2. Primers used for RT-PCR**

| Gene | Primer sequences |
| --- | --- |
| *GAPDH*-Forward | GGAGCGAGATCCCTCCAAAAT |
| *GAPDH* -Reverse | GGCTGTTGTCATACTTCTCATGG |
| *HOXB-AS4-*Forward | AACCACTACTACCCAGGCCT |
| *HOXB-AS4*-Reverse | CTTCGTTTTGGTTCGGAGGC |
| *LINC00524*-Forward | CCAGCTCCATGATGATGCCT |
| *LINC00524-*Reverse | AGATGCTTGTGGACACCTGG |
| *LGALS8-AS1*-Forward | GCTGGGACTTTTCAGGACCA |
| *LGALS8-AS1*-Reverse | TGAGAAAACCCTGGCGTTGA |
| *KCNJ2-AS1*-Forward | AAGCCACTGAGTCAATGGGG |
| *KCNJ2-AS1*-Reverse | TCGGGAGAACAAACAGCCAA |
| *LINC02580*-Forward | AGCCCTAGAAGTCCGTGAGT |
| *LINC02580*-Reverse | GAATCTGGGTTGGAAGGCCA |
| *LINC00645*-Forward | CAGAGGTGGTGCCTTGACAT |
| *LINC00645*-Reverse | ATATCCTCTGTGGCCCATGC |
| *LINC01929*-Forward | TGCCTTTGGTGTGGTCCTGTTTC |
| *LINC01929* -Reverse | AAGATGCCCATACCAGACCTCCAG |

**Table S3. Primers used for RNA pull-down assay**

| Gene | Primer sequences |
| --- | --- |
| T7-*LINC00524*-Forward | TAATACGACTCACTATAGGGGGTTCACGCGATTCTCCTG |
| T7-*LINC00524*-Reverse | GTTGTTACTCTCATTTATTTGCTAAGTTTCC |
| T7-antisense *LINC00524*-Forward | GGTTCACGCGATTCTCCTG |
| T7-antisense *LINC00524*-Reverse | TAATACGACTCACTATAGGGGTTGTTACTCTCATTTATTTGCTAAGTTTCC |

**Table S4. Primers used for RIP-qPCR assay**

| Gene | Primer sequences (5’-3’) |
| --- | --- |
| *LINC00524*-Forward | AGAGACCTCCTGACCTCCTG |
| *LINC00524*-Reverse | TGAAAGAGGAGATGCGTGGG |

**Table S5. 360 significantly different lncRNAs were identified in BC and adjacent normal tissues across the TCGA and GEO databases**

| id | logFC.x | t | P.Value | adj.P.Val | B | conMean | logFC.y | pValue | fdr |
| --- | --- | --- | --- | --- | --- | --- | --- | --- | --- |
| LINC00524 | 1.360793 | 4.99914229 | 1.80E-05 | 0.00023871 | 2.69818872 | 0.02153629 | 2.85709859 | 3.81E-08 | 7.17E-08 |
| AC002310.2 | 1.31721215 | 4.45013244 | 9.06E-05 | 0.00071498 | 1.13601131 | 0.01076239 | 1.33743153 | 1.73E-12 | 4.35E-12 |
| AC002480.1 | -1.4251997 | -2.6166196 | 0.01324454 | 0.03079783 | -3.5693454 | 0.20767459 | -1.4682411 | 5.93E-34 | 6.07E-33 |
| AC002551.1 | 1.12891636 | 2.96415133 | 0.0055678 | 0.01573088 | -2.7740319 | 0.0217052 | 1.67309721 | 0.0004803 | 0.00065763 |
| AC003965.1 | -1.4020634 | -3.1031501 | 0.00388544 | 0.01189248 | -2.4397613 | 0.29369722 | 1.07438245 | 7.53E-10 | 1.60E-09 |
| AC003986.2 | -2.0819519 | -5.10446 | 1.32E-05 | 0.00019307 | 3.00099129 | 0.8645362 | -3.0789751 | 1.68E-47 | 4.82E-46 |
| AC004490.1 | 1.1629796 | 2.47471733 | 0.01858643 | 0.03996245 | -3.8756248 | 0.20725169 | -2.9225689 | 1.80E-38 | 2.49E-37 |
| AC004884.2 | 1.60567762 | 8.02518099 | 2.71E-09 | 4.74E-07 | 11.2824907 | 0.10492801 | 1.8597839 | 7.64E-06 | 1.21E-05 |
| AC005544.1 | 1.48906577 | 3.8792495 | 0.00046752 | 0.00232097 | -0.4393661 | 0.12801798 | 1.64272286 | 7.26E-08 | 1.34E-07 |
| AC005725.1 | -2.1644905 | -3.5136796 | 0.0012937 | 0.00513797 | -1.4067817 | 0.2128378 | -1.105386 | 3.43E-23 | 1.71E-22 |
| AC007336.1 | -1.5175769 | -5.3150366 | 7.07E-06 | 0.00012788 | 3.60818311 | 0.19017617 | -1.3599134 | 9.87E-34 | 9.91E-33 |
| AC007342.4 | 1.41989981 | 5.29878331 | 7.42E-06 | 0.0001326 | 3.56125259 | 0.29899186 | 1.10685475 | 3.42E-05 | 5.18E-05 |
| AC007541.1 | -1.3308357 | -3.5339323 | 0.00122381 | 0.00489626 | -1.3542476 | 1.32044344 | -1.09572 | 4.00E-45 | 9.50E-44 |
| AC007637.1 | 1.06480653 | 3.06311073 | 0.00431277 | 0.01296485 | -2.5369241 | 1.03827759 | -1.1449898 | 4.36E-33 | 4.22E-32 |
| AC007938.1 | -2.349941 | -3.8902157 | 0.00045325 | 0.00227792 | -0.4097712 | 0.28724458 | -2.2601877 | 2.72E-58 | 2.56E-56 |
| AC008115.2 | 1.06926173 | 3.05424836 | 0.0044132 | 0.01318565 | -2.5583362 | 0.0360208 | 1.45009725 | 9.19E-06 | 1.45E-05 |
| AC008676.1 | 1.17813249 | 3.82855338 | 0.00053937 | 0.00261301 | -0.5757804 | 0.03362115 | 1.13640533 | 8.93E-08 | 1.63E-07 |
| AC008780.1 | -2.4380274 | -4.6758235 | 4.68E-05 | 0.00044999 | 1.77415654 | 0.13609008 | -2.5316485 | 1.61E-61 | 2.59E-59 |
| AC009063.2 | 1.52774508 | 4.21828074 | 0.0001775 | 0.00115459 | 0.48846506 | 0.01500296 | 2.13612385 | 0.00338556 | 0.00431678 |
| AC009142.1 | 1.66950844 | 5.20601503 | 9.77E-06 | 0.00015959 | 3.29357616 | 0.0912756 | -2.7224088 | 2.84E-47 | 7.90E-46 |
| AC009549.1 | -1.3297037 | -4.4546024 | 8.94E-05 | 0.00070916 | 1.14858193 | 1.31288836 | -1.4706214 | 8.08E-28 | 5.37E-27 |
| AC010207.1 | -1.5576906 | -4.1707502 | 0.00020355 | 0.00127974 | 0.35690658 | 0.38816556 | -1.9625072 | 6.73E-46 | 1.70E-44 |
| AC010255.2 | 1.02017236 | 2.80194964 | 0.00839481 | 0.02159485 | -3.1527947 | 0.0521618 | 1.31523785 | 2.29E-06 | 3.78E-06 |
| AC010307.2 | 1.74427172 | 3.29006985 | 0.00237016 | 0.00807409 | -1.9773632 | 0.02738652 | 1.73228067 | 3.34E-12 | 8.21E-12 |
| AC010531.3 | 1.24732489 | 4.06365267 | 0.00027677 | 0.00159475 | 0.06214971 | 0.22136714 | -1.1740658 | 2.61E-19 | 1.00E-18 |
| AC012368.2 | -1.3963576 | -4.0558752 | 0.00028299 | 0.00161308 | 0.04083913 | 0.15891363 | -1.1562731 | 2.64E-17 | 8.85E-17 |
| AC012417.1 | -2.1527478 | -6.3859241 | 2.97E-07 | 1.41E-05 | 6.69888481 | 0.08081514 | -1.2480759 | 3.11E-27 | 1.99E-26 |
| AC012462.1 | 2.36350012 | 5.9844061 | 9.70E-07 | 3.20E-05 | 5.54317221 | 0.00198341 | 4.69485883 | 2.93E-31 | 2.46E-30 |
| AC012668.3 | 1.28366327 | 3.89049571 | 0.00045289 | 0.00227792 | -0.4090151 | 0.0041253 | 2.43877421 | 5.33E-08 | 9.93E-08 |
| AC013275.1 | 1.33647806 | 3.82806446 | 0.00054011 | 0.00261301 | -0.5770927 | 0.19530012 | -2.7252995 | 7.04E-37 | 8.77E-36 |
| AC013652.1 | 1.04112933 | 3.57562638 | 0.00109124 | 0.00449089 | -1.2456759 | 0.0540146 | 3.2278418 | 6.36E-27 | 3.95E-26 |
| AC015712.2 | -1.0637795 | -4.0367413 | 0.00029889 | 0.00166428 | -0.0115317 | 1.08777402 | -1.5517297 | 3.61E-38 | 4.89E-37 |
| AC015908.2 | -3.7527741 | -10.915861 | 1.51E-12 | 1.84E-09 | 18.5391198 | 0.84598989 | -2.0793021 | 8.81E-41 | 1.50E-39 |
| AC015908.3 | 1.06527111 | 3.88795213 | 0.00045616 | 0.00228315 | -0.4158824 | 0.78687146 | -2.1927604 | 5.30E-63 | 1.20E-60 |
| AC015909.1 | 1.11565286 | 2.99592859 | 0.00513137 | 0.01487681 | -2.6983737 | 0.02434326 | 1.51413352 | 3.42E-06 | 5.56E-06 |
| AC016571.1 | 1.31808485 | 4.8171254 | 3.09E-05 | 0.00033291 | 2.17679434 | 0.02129951 | 1.32257078 | 1.09E-08 | 2.13E-08 |
| AC016924.1 | -1.3579712 | -3.1845477 | 0.00313745 | 0.01007887 | -2.240142 | 0.98837859 | -3.6638549 | 2.76E-57 | 2.31E-55 |
| AC018450.1 | -2.149486 | -4.456207 | 8.90E-05 | 0.00070815 | 1.15309498 | 0.1895861 | -1.3832634 | 1.21E-32 | 1.12E-31 |
| AC020659.1 | -1.3086333 | -2.4597253 | 0.01925331 | 0.04133615 | -3.9073073 | 0.84097065 | -1.0795646 | 1.04E-17 | 3.57E-17 |
| AC021087.3 | 1.22465947 | 2.83174041 | 0.00779106 | 0.02036347 | -3.0841868 | 0.47435409 | 1.58383468 | 9.09E-09 | 1.79E-08 |
| AC021188.1 | -2.0349427 | -4.2348669 | 0.00016921 | 0.00111698 | 0.53447554 | 0.67403162 | -1.5531792 | 1.54E-47 | 4.44E-46 |
| AC022164.1 | 2.01098971 | 3.82154324 | 0.00055012 | 0.0026424 | -0.5945906 | 0.00556259 | 2.89215268 | 1.83E-21 | 8.10E-21 |
| AC022211.3 | 1.50056675 | 4.08890016 | 0.00025747 | 0.00151537 | 0.13141942 | 0.20594408 | 1.5512347 | 1.71E-25 | 9.68E-25 |
| AC022706.1 | -1.2156869 | -4.1599866 | 0.00020995 | 0.00130998 | 0.327176 | 2.08392793 | -1.1271284 | 2.07E-40 | 3.40E-39 |
| AC023908.3 | 1.36110012 | 3.77379747 | 0.00062905 | 0.00293495 | -0.7223549 | 0.12885963 | 1.2455902 | 4.77E-15 | 1.39E-14 |
| AC023983.2 | 1.29652939 | 2.51398842 | 0.01693848 | 0.03722199 | -3.7920117 | 0.1460239 | 1.24697358 | 5.58E-05 | 8.26E-05 |
| AC025165.1 | 1.34098825 | 3.58798488 | 0.00105468 | 0.0043699 | -1.2133875 | 0.41225905 | -1.2660537 | 1.14E-29 | 8.45E-29 |
| AC026992.1 | 1.04944738 | 2.9088204 | 0.00641233 | 0.01753876 | -2.9046534 | 0.0225128 | 1.24423372 | 0.00255035 | 0.00328885 |
| AC034199.1 | 1.3106454 | 3.01902942 | 0.0048346 | 0.01419291 | -2.6430856 | 0.01278137 | 1.84825756 | 4.77E-24 | 2.50E-23 |
| AC036108.2 | -1.8996673 | -5.8460455 | 1.46E-06 | 4.08E-05 | 5.14349813 | 0.68671818 | -3.381484 | 2.26E-50 | 9.02E-49 |
| AC036108.3 | -3.2604399 | -4.8235922 | 3.03E-05 | 0.00032807 | 2.1952699 | 2.3171208 | -2.4777206 | 4.32E-50 | 1.68E-48 |
| AC040970.1 | 1.16128862 | 3.90739285 | 0.00043175 | 0.00220157 | -0.3633537 | 0.65345015 | 1.19654299 | 1.18E-20 | 4.95E-20 |
| AC048382.2 | -3.3759561 | -4.8265021 | 3.00E-05 | 0.00032694 | 2.20358449 | 0.9746915 | -1.4769443 | 9.27E-34 | 9.35E-33 |
| AC055874.1 | -1.2076417 | -4.451565 | 9.02E-05 | 0.00071375 | 1.14003965 | 0.33316248 | -2.2949495 | 6.22E-67 | 2.93E-64 |
| AC068987.1 | -2.2709683 | -4.4333941 | 9.51E-05 | 0.00073996 | 1.08896641 | 0.31550089 | -1.5741044 | 5.34E-38 | 7.17E-37 |
| AC073359.1 | -2.3384353 | -4.6402186 | 5.20E-05 | 0.00048715 | 1.67304798 | 0.30998526 | -3.3938669 | 9.56E-68 | 5.85E-65 |
| AC073370.1 | 1.54169035 | 5.85041794 | 1.44E-06 | 4.05E-05 | 5.15613564 | 0.04123164 | 1.6460204 | 8.88E-16 | 2.70E-15 |
| AC078909.1 | -2.6303396 | -8.1135723 | 2.12E-09 | 4.33E-07 | 11.5212537 | 0.04329329 | -1.7732113 | 6.60E-17 | 2.16E-16 |
| AC084064.1 | -1.8164057 | -4.205203 | 0.00018432 | 0.00119419 | 0.45222405 | 0.46499705 | -3.7562409 | 2.08E-69 | 1.59E-66 |
| AC087521.1 | 1.03628566 | 4.14234974 | 0.00022087 | 0.00136137 | 0.27851091 | 0.21875226 | -1.4449723 | 1.14E-16 | 3.68E-16 |
| AC090371.2 | 1.30501953 | 4.47946082 | 8.32E-05 | 0.00067601 | 1.21854331 | 0.00929747 | -1.094114 | 4.08E-22 | 1.89E-21 |
| AC090844.2 | -1.6740706 | -4.1167205 | 0.00023774 | 0.00142672 | 0.20790565 | 0.94321571 | -1.6734477 | 1.40E-29 | 1.03E-28 |
| AC092164.1 | -4.1572791 | -10.915521 | 1.51E-12 | 1.84E-09 | 18.5383339 | 2.36401125 | -2.204292 | 2.31E-42 | 4.31E-41 |
| AC092198.1 | 2.28187697 | 3.58135519 | 0.00107414 | 0.004428 | -1.2307146 | 0.01489755 | 3.25104477 | 5.10E-18 | 1.79E-17 |
| AC092757.2 | 1.08714775 | 3.19816344 | 0.00302656 | 0.00977826 | -2.206484 | 0.3432692 | -1.0293937 | 6.16E-22 | 2.82E-21 |
| AC093297.2 | -3.0416762 | -3.4941101 | 0.00136487 | 0.0053339 | -1.4574147 | 4.63138804 | 1.75030821 | 9.95E-08 | 1.82E-07 |
| AC093904.4 | 2.98619962 | 3.7262074 | 0.00071862 | 0.00326556 | -0.8490773 | 0.07534909 | 2.62419499 | 1.87E-07 | 3.36E-07 |
| AC096564.1 | -2.743604 | -7.4853071 | 1.24E-08 | 1.35E-06 | 9.80357781 | 0.07994927 | -1.1698009 | 7.17E-57 | 5.69E-55 |
| AC096564.2 | -1.8083971 | -3.6330802 | 0.00093107 | 0.00396538 | -1.0951643 | 0.03677599 | -1.2840974 | 2.18E-25 | 1.22E-24 |
| AC096921.2 | -1.976179 | -6.1570132 | 5.83E-07 | 2.30E-05 | 6.04090365 | 1.53318099 | -2.2112467 | 2.62E-59 | 2.91E-57 |
| AC103706.1 | 2.14649013 | 3.88905115 | 0.00045475 | 0.00227914 | -0.4129154 | 0.50725507 | 1.82953948 | 1.20E-32 | 1.11E-31 |
| AC103740.2 | -1.1469479 | -4.6988527 | 4.37E-05 | 0.0004254 | 1.83963192 | 0.12364258 | 1.41785757 | 0.00064709 | 0.00087737 |
| AC103760.1 | -2.2429008 | -4.8262732 | 3.01E-05 | 0.00032694 | 2.20293041 | 0.95840273 | 1.21539293 | 4.15E-11 | 9.53E-11 |
| AC104393.1 | -3.7730149 | -5.221439 | 9.33E-06 | 0.00015665 | 3.33805682 | 0.27000771 | -1.4842375 | 3.68E-27 | 2.34E-26 |
| AC105046.1 | -1.9409294 | -6.0434434 | 8.15E-07 | 2.80E-05 | 5.71353863 | 0.44187292 | -1.6828536 | 3.51E-25 | 1.96E-24 |
| AC107959.1 | -1.8565733 | -4.386023 | 0.00010919 | 0.00081935 | 0.95605973 | 0.79936114 | -1.7725217 | 1.97E-62 | 3.84E-60 |
| AC108134.3 | -1.4985437 | -5.1275802 | 1.23E-05 | 0.00018462 | 3.06755442 | 1.33462519 | -1.5097666 | 2.65E-44 | 5.82E-43 |
| AC108156.1 | -1.5756854 | -2.8769608 | 0.00695183 | 0.01863211 | -2.9792088 | 0.23222628 | -2.5868405 | 1.10E-48 | 3.50E-47 |
| AC110597.1 | -1.6893533 | -4.5208791 | 7.37E-05 | 0.00061536 | 1.33530974 | 0.36936581 | -1.6566674 | 1.11E-44 | 2.53E-43 |
| AC112484.3 | -1.0108931 | -2.6587534 | 0.01195585 | 0.02861554 | -3.4762425 | 0.35368168 | 1.22801566 | 2.14E-15 | 6.38E-15 |
| AC114956.2 | 1.24518991 | 5.13587592 | 1.20E-05 | 0.00018417 | 3.09144518 | 0.16755097 | 1.57976533 | 0.00567786 | 0.00709762 |
| AC119424.1 | 1.02733785 | 2.54338951 | 0.01579364 | 0.03524529 | -3.7288311 | 0.7677912 | -1.2446955 | 2.36E-32 | 2.14E-31 |
| AC120193.1 | 1.33052399 | 3.49107059 | 0.00137625 | 0.00536552 | -1.4652674 | 0.01656894 | 1.5976802 | 0.00143687 | 0.00189201 |
| AC124067.3 | 1.26793041 | 3.50827524 | 0.00131298 | 0.00520167 | -1.4207773 | 0.01263606 | 2.36616735 | 1.84E-06 | 3.05E-06 |
| AC124312.2 | 1.04580053 | 2.55427629 | 0.01538808 | 0.03451368 | -3.7053114 | 1.38052022 | -1.0492036 | 7.98E-27 | 4.91E-26 |
| AC130651.1 | 1.41856057 | 4.040384 | 0.0002958 | 0.00165518 | -0.0015677 | 0.06551077 | 1.21701715 | 6.54E-05 | 9.63E-05 |
| AC131009.2 | 1.36252857 | 4.68383297 | 4.57E-05 | 0.00044213 | 1.79692178 | 0.02434178 | 2.47030324 | 4.68E-17 | 1.55E-16 |
| AC132192.2 | 2.38177125 | 4.04989739 | 0.00028787 | 0.00163458 | 0.02446872 | 0.51389118 | 1.10456609 | 2.64E-21 | 1.15E-20 |
| AC134312.1 | -1.3250554 | -3.650439 | 0.00088733 | 0.00381232 | -1.0494896 | 0.10767887 | -1.3800235 | 1.48E-17 | 5.06E-17 |
| AC134312.5 | 2.23908317 | 6.37969097 | 3.03E-07 | 1.42E-05 | 6.68100662 | 0.08842448 | 3.25473306 | 3.36E-50 | 1.32E-48 |
| AC136443.3 | -1.2434555 | -3.1570578 | 0.00337325 | 0.01065178 | -2.3078665 | 0.00954388 | 1.38713217 | 1.50E-07 | 2.71E-07 |
| AC136475.5 | -1.8249161 | -5.9730669 | 1.00E-06 | 3.27E-05 | 5.51043707 | 0.21247046 | -1.0245137 | 1.66E-24 | 8.90E-24 |
| AC138904.1 | 1.8256579 | 2.59367351 | 0.01399922 | 0.03206075 | -3.6196405 | 0.11090975 | 2.50489258 | 1.40E-37 | 1.85E-36 |
| AC140847.2 | -2.8124416 | -5.6649082 | 2.50E-06 | 6.20E-05 | 4.61971412 | 0.19990391 | -1.1843773 | 1.22E-25 | 6.96E-25 |
| AC145124.1 | -2.5202509 | -6.324488 | 3.56E-07 | 1.54E-05 | 6.52257006 | 0.38846301 | -1.0106929 | 2.04E-17 | 6.88E-17 |
| AC147651.2 | -5.0723173 | -15.229393 | 1.40E-16 | 6.85E-13 | 27.2860518 | 0.33010504 | -3.5022295 | 6.52E-61 | 9.27E-59 |
| AC233976.1 | 1.02977152 | 3.11764919 | 0.00374086 | 0.01156291 | -2.4044069 | 0.18534814 | -1.4512357 | 1.17E-20 | 4.90E-20 |
| AC245014.1 | 1.7075681 | 5.28172831 | 7.80E-06 | 0.00013774 | 3.51201692 | 0.05589424 | 1.31795786 | 1.91E-06 | 3.16E-06 |
| ADAMTS9-AS2 | -2.8093736 | -10.190153 | 8.94E-12 | 5.46E-09 | 16.8272878 | 0.78329783 | -3.0735367 | 1.34E-61 | 2.22E-59 |
| AF001548.1 | 1.00211091 | 3.15161257 | 0.00342191 | 0.01076371 | -2.3212443 | 0.34120041 | -1.3273533 | 4.53E-23 | 2.24E-22 |
| AGAP1-IT1 | 2.42747323 | 5.01037291 | 1.74E-05 | 0.00023473 | 2.73044374 | 0.16892816 | 2.70996267 | 4.40E-37 | 5.56E-36 |
| AJ011932.1 | -1.59167 | -2.6064995 | 0.01357263 | 0.03139312 | -3.5915633 | 0.38110541 | -1.4158959 | 3.05E-26 | 1.81E-25 |
| AL021026.1 | -1.6352131 | -3.4448199 | 0.00156124 | 0.0059259 | -1.5843718 | 0.30391151 | -1.2463023 | 4.96E-36 | 5.75E-35 |
| AL021408.1 | -2.6473239 | -6.7127837 | 1.14E-07 | 7.06E-06 | 7.63277552 | 0.35608961 | -1.8407891 | 8.21E-49 | 2.67E-47 |
| AL022329.1 | -1.213639 | -4.0305871 | 0.00030419 | 0.00167973 | -0.028359 | 0.27167294 | -2.4561422 | 4.77E-45 | 1.12E-43 |
| AL023803.1 | 1.60955156 | 2.71729791 | 0.01035739 | 0.02556638 | -3.3452946 | 0.15722994 | 1.58000912 | 2.22E-13 | 5.86E-13 |
| AL031283.2 | 1.3233409 | 3.74769353 | 0.00067674 | 0.00310199 | -0.7919429 | 0.00815488 | -1.2029214 | 4.03E-12 | 9.86E-12 |
| AL031985.3 | -2.38068 | -3.1879315 | 0.00310953 | 0.01003308 | -2.2317843 | 0.88302888 | 1.12214245 | 4.25E-34 | 4.42E-33 |
| AL049775.3 | -1.5064704 | -3.1671553 | 0.00328474 | 0.010433 | -2.2830262 | 0.04258689 | -1.4413946 | 8.24E-13 | 2.11E-12 |
| AL049838.1 | -1.9320221 | -3.6054193 | 0.00100512 | 0.00421256 | -1.1677559 | 1.7981793 | -1.1421919 | 1.42E-38 | 1.98E-37 |
| AL050327.1 | -2.2948591 | -8.3095917 | 1.24E-09 | 3.02E-07 | 12.0471906 | 0.35320459 | -1.3231979 | 7.73E-22 | 3.52E-21 |
| AL096828.1 | 1.81373324 | 3.37974626 | 0.00186245 | 0.00676436 | -1.750688 | 0.04374923 | 1.06930483 | 0.00351299 | 0.0044746 |
| AL109615.2 | 1.25074032 | 3.29691229 | 0.00232717 | 0.00797209 | -1.9601737 | 0.01021815 | 1.58673935 | 3.16E-05 | 4.80E-05 |
| AL109811.1 | -2.9277785 | -7.68237 | 7.08E-09 | 1.00E-06 | 10.3473903 | 0.33009571 | -1.8275794 | 1.74E-31 | 1.49E-30 |
| AL109924.2 | 1.05749541 | 2.54166424 | 0.01585881 | 0.03535172 | -3.7325522 | 0.02577944 | 2.12525715 | 2.00E-20 | 8.26E-20 |
| AL118508.2 | 1.09854542 | 2.36060242 | 0.02424046 | 0.04952515 | -4.1134063 | 0.09854056 | -1.803128 | 2.01E-32 | 1.84E-31 |
| AL121832.1 | 1.14656707 | 3.45414231 | 0.00152212 | 0.00580904 | -1.5604237 | 0.0413341 | 1.78631084 | 3.19E-16 | 9.96E-16 |
| AL121894.1 | -1.4528174 | -3.7093979 | 0.00075313 | 0.00338458 | -0.8936843 | 0.12609715 | -2.4259864 | 8.90E-49 | 2.85E-47 |
| AL122008.3 | -3.7983518 | -6.2906649 | 3.93E-07 | 1.66E-05 | 6.42540975 | 0.39955659 | -1.7336459 | 5.77E-30 | 4.33E-29 |
| AL135960.1 | 1.61604922 | 4.55597701 | 6.65E-05 | 0.00058011 | 1.43444518 | 0.15841065 | -2.060481 | 1.99E-46 | 5.14E-45 |
| AL136366.1 | -1.7648277 | -5.1790754 | 1.06E-05 | 0.00016891 | 3.21591163 | 0.84795141 | -1.2787631 | 3.43E-40 | 5.57E-39 |
| AL136369.2 | -1.4722305 | -2.92139 | 0.00621051 | 0.01715 | -2.875106 | 0.06966162 | -2.5999171 | 7.66E-57 | 6.01E-55 |
| AL137026.1 | -3.2485021 | -5.0375841 | 1.61E-05 | 0.00022207 | 2.80863149 | 0.48335656 | -1.7230121 | 4.60E-51 | 1.93E-49 |
| AL157373.2 | 1.80763298 | 4.71831511 | 4.13E-05 | 0.0004109 | 1.89501255 | 0.11012988 | 1.23418003 | 0.00073211 | 0.00098934 |
| AL158055.1 | -3.0867618 | -8.4434404 | 8.59E-10 | 2.21E-07 | 12.4034512 | 0.21697282 | -1.9272331 | 1.93E-32 | 1.77E-31 |
| AL158212.3 | -2.3606819 | -3.7024487 | 0.00076786 | 0.00343498 | -0.9121013 | 2.18012668 | -1.0843848 | 3.06E-47 | 8.46E-46 |
| AL160408.3 | 1.10448727 | 3.42199789 | 0.00166111 | 0.00622279 | -1.6428709 | 0.01115791 | 2.67632705 | 0.00068363 | 0.00092526 |
| AL160408.4 | 1.71687391 | 5.24385311 | 8.73E-06 | 0.00014861 | 3.4027141 | 0.03380521 | 2.74243276 | 6.88E-05 | 0.00010113 |
| AL162171.1 | -4.0493849 | -8.9532536 | 2.18E-10 | 6.25E-08 | 13.7384456 | 1.04167403 | -1.2366629 | 2.93E-36 | 3.45E-35 |
| AL162408.1 | -1.8709635 | -4.9574137 | 2.04E-05 | 0.00025619 | 2.57842081 | 0.06821036 | -1.1801779 | 1.31E-39 | 2.01E-38 |
| AL163051.1 | -1.2353937 | -2.8547603 | 0.00735264 | 0.019403 | -3.0308715 | 0.26456756 | 1.07619918 | 4.23E-22 | 1.96E-21 |
| AL353693.1 | -5.9508284 | -7.449704 | 1.37E-08 | 1.45E-06 | 9.70486141 | 1.42565043 | -3.3545154 | 1.75E-47 | 4.97E-46 |
| AL356215.1 | 1.04948598 | 2.72183859 | 0.01024211 | 0.0253202 | -3.3350628 | 0.18496875 | 1.14630775 | 0.00072928 | 0.00098596 |
| AL356489.2 | -3.55073 | -8.1807809 | 1.76E-09 | 3.90E-07 | 11.702136 | 0.69860676 | -3.059122 | 3.41E-60 | 4.54E-58 |
| AL359715.2 | -1.5667381 | -4.8453632 | 2.84E-05 | 0.0003168 | 2.25749745 | 0.52919426 | -1.0313773 | 1.37E-29 | 1.01E-28 |
| AL359962.1 | 1.52081543 | 6.05297991 | 7.92E-07 | 2.76E-05 | 5.74104699 | 1.41075052 | -1.0447178 | 2.66E-34 | 2.79E-33 |
| AL359979.2 | 1.57191693 | 5.34321351 | 6.50E-06 | 0.00012065 | 3.68956216 | 0.05349548 | 1.51186791 | 8.07E-10 | 1.71E-09 |
| AL391361.2 | 1.61291068 | 3.81166769 | 0.00056561 | 0.00269561 | -0.6210673 | 0.08061566 | -2.2870239 | 8.88E-32 | 7.77E-31 |
| AL391807.1 | -2.1341638 | -7.104183 | 3.68E-08 | 2.99E-06 | 8.73983531 | 0.10978682 | -1.9102595 | 1.87E-51 | 8.28E-50 |
| AL391845.2 | 1.73142787 | 5.91811049 | 1.18E-06 | 3.58E-05 | 5.35173011 | 0.02134826 | 2.60442373 | 1.56E-48 | 4.88E-47 |
| AL391883.1 | -3.7811214 | -6.0601511 | 7.76E-07 | 2.76E-05 | 5.76173042 | 0.65968576 | -4.2317984 | 4.25E-60 | 5.53E-58 |
| AL445183.2 | 1.11588031 | 2.59390329 | 0.01399147 | 0.03205831 | -3.6191383 | 0.03703775 | -1.0437226 | 3.10E-14 | 8.62E-14 |
| AL445307.1 | -2.2553769 | -7.3581024 | 1.78E-08 | 1.81E-06 | 9.45024159 | 0.20673004 | -2.4448455 | 4.59E-43 | 8.97E-42 |
| AL449423.1 | 1.02472785 | 3.47245358 | 0.00144802 | 0.00556976 | -1.5132975 | 0.01498124 | 2.70801074 | 2.35E-14 | 6.59E-14 |
| AL589765.1 | 1.05918797 | 3.60519024 | 0.00100575 | 0.00421256 | -1.168356 | 0.0322974 | 1.73826694 | 4.76E-11 | 1.09E-10 |
| AL591242.1 | -1.6413118 | -6.0443122 | 8.13E-07 | 2.80E-05 | 5.71604478 | 0.05592083 | -1.501968 | 4.93E-21 | 2.11E-20 |
| AL606970.1 | 1.33118978 | 3.93418142 | 0.00040019 | 0.00207652 | -0.2908174 | 0.02253937 | -3.3928016 | 1.19E-65 | 4.06E-63 |
| AL645634.2 | -2.1629424 | -3.812505 | 0.00056428 | 0.0026919 | -0.6188233 | 0.05054646 | -1.0338686 | 6.89E-16 | 2.11E-15 |
| AL672277.1 | -2.2851622 | -3.1299189 | 0.00362251 | 0.01127356 | -2.3744192 | 0.22143678 | -1.4839693 | 2.90E-19 | 1.11E-18 |
| AP000251.1 | 1.42901688 | 4.82364484 | 3.03E-05 | 0.00032807 | 2.19542018 | 0.1662714 | 2.46319907 | 3.18E-37 | 4.08E-36 |
| AP000442.1 | -1.1872424 | -4.0635647 | 0.00027684 | 0.00159475 | 0.06190849 | 0.1609029 | -1.4323531 | 5.75E-30 | 4.32E-29 |
| AP000695.2 | 2.87426549 | 6.08167844 | 7.28E-07 | 2.67E-05 | 5.82380867 | 0.14853384 | 1.46659584 | 3.12E-28 | 2.13E-27 |
| AP000696.1 | 3.01847297 | 5.10660181 | 1.31E-05 | 0.00019242 | 3.00715626 | 0.0193206 | 4.33250636 | 6.12E-19 | 2.29E-18 |
| AP000757.1 | 1.14912257 | 4.14689873 | 0.000218 | 0.00134975 | 0.29105684 | 0.34812626 | -1.3634661 | 5.58E-32 | 4.96E-31 |
| AP000866.5 | -1.4504652 | -3.7618425 | 0.00065047 | 0.00300294 | -0.7542479 | 0.23806769 | -1.2552673 | 5.73E-25 | 3.14E-24 |
| AP001107.5 | -1.3883685 | -5.1444549 | 1.17E-05 | 0.00018068 | 3.11615543 | 0.95335349 | -1.6201147 | 5.25E-34 | 5.41E-33 |
| AP001189.5 | 1.80591516 | 5.82071721 | 1.58E-06 | 4.25E-05 | 5.0702856 | 0.08671677 | -1.5729175 | 7.92E-22 | 3.60E-21 |
| AP001453.2 | -1.0190993 | -3.2042019 | 0.00297858 | 0.00965519 | -2.191533 | 0.61100489 | 1.80013079 | 3.44E-35 | 3.79E-34 |
| AP001528.1 | -1.8689458 | -5.2479441 | 8.63E-06 | 0.00014785 | 3.41451748 | 0.90827756 | -1.1348677 | 2.05E-34 | 2.16E-33 |
| AP001922.3 | 1.44890306 | 3.91444382 | 0.00042322 | 0.00217036 | -0.3442787 | 0.14917798 | -2.4540573 | 7.84E-29 | 5.50E-28 |
| AP002026.1 | -2.1768177 | -6.8119666 | 8.56E-08 | 5.50E-06 | 7.91457415 | 0.60947273 | -1.748101 | 1.40E-52 | 7.16E-51 |
| AP002358.1 | 1.21081816 | 3.23794929 | 0.00272353 | 0.00901479 | -2.1077067 | 0.0139286 | 1.66114661 | 0.02259162 | 0.02658937 |
| AP003032.1 | -2.6943988 | -5.3434147 | 6.50E-06 | 0.00012065 | 3.69014326 | 0.66015377 | -1.5403526 | 3.30E-24 | 1.74E-23 |
| AP003071.4 | -2.5530355 | -5.1322257 | 1.22E-05 | 0.00018444 | 3.08093262 | 0.92358009 | -2.2019067 | 2.55E-52 | 1.24E-50 |
| AP003110.1 | 1.50731575 | 5.38476597 | 5.75E-06 | 0.00010881 | 3.8096132 | 0.16643088 | -1.2049533 | 1.16E-25 | 6.63E-25 |
| AP003548.1 | 1.08280053 | 3.19984574 | 0.00301312 | 0.00975419 | -2.2023202 | 0.16997583 | -1.5674729 | 2.29E-18 | 8.22E-18 |
| AP003680.1 | 1.01968358 | 4.03975159 | 0.00029633 | 0.00165627 | -0.0032977 | 0.00843822 | 1.70829731 | 4.48E-12 | 1.09E-11 |
| AP003721.1 | 1.56253304 | 5.5035822 | 4.04E-06 | 8.77E-05 | 4.15309783 | 0.02834595 | 2.08830591 | 2.42E-16 | 7.61E-16 |
| AP005264.1 | -2.7900817 | -9.3971471 | 6.76E-11 | 2.54E-08 | 14.8717151 | 0.89371542 | -3.1716972 | 5.67E-61 | 8.47E-59 |
| AP005432.1 | -1.1871923 | -2.8665279 | 0.00713756 | 0.0189496 | -3.0035168 | 0.01478462 | 1.74367344 | 0.00868789 | 0.01063282 |
| APCDD1L-DT | -2.8588743 | -9.3659265 | 7.34E-11 | 2.56E-08 | 14.7929084 | 1.93393176 | -2.2985469 | 1.03E-39 | 1.61E-38 |
| ARHGAP5-AS1 | -2.2807675 | -3.7546966 | 0.00066361 | 0.00304963 | -0.7732927 | 3.0416034 | -1.163348 | 9.19E-44 | 1.93E-42 |
| ATP2A1-AS1 | 1.59847764 | 4.6187492 | 5.53E-05 | 0.00050521 | 1.61215379 | 0.31482525 | 2.20462146 | 6.56E-43 | 1.26E-41 |
| ATP2B1-AS1 | -3.1099098 | -6.4092718 | 2.77E-07 | 1.34E-05 | 6.76583154 | 0.8910294 | -1.069692 | 1.46E-38 | 2.03E-37 |
| B4GALT1-AS1 | -1.4559882 | -4.3977052 | 0.00010554 | 0.00080433 | 0.98880297 | 2.9837977 | -1.5097586 | 6.49E-37 | 8.16E-36 |
| BBOX1-AS1 | 1.06154436 | 4.33865671 | 0.0001253 | 0.0008969 | 0.8235283 | 0.09119478 | 1.31692495 | 5.11E-05 | 7.60E-05 |
| BVES-AS1 | -1.2292743 | -4.3868672 | 0.00010892 | 0.00081935 | 0.9584252 | 0.15156441 | -1.5449938 | 1.95E-43 | 3.92E-42 |
| BX255923.1 | -3.7275784 | -10.028366 | 1.34E-11 | 6.56E-09 | 16.4355374 | 0.04785598 | -1.1656394 | 3.89E-22 | 1.80E-21 |
| BX255925.1 | 1.01963832 | 2.79833533 | 0.00847096 | 0.02173096 | -3.1610883 | 0.08566816 | 1.05702409 | 2.89E-13 | 7.60E-13 |
| BX324167.1 | 2.18804504 | 3.04094808 | 0.00456808 | 0.01354892 | -2.590406 | 0.04134854 | 2.74738374 | 1.29E-19 | 5.03E-19 |
| BX664727.3 | -2.3521067 | -5.3958168 | 5.56E-06 | 0.00010612 | 3.84154813 | 0.08818114 | -1.2897907 | 1.58E-27 | 1.03E-26 |
| CARMN | -1.4590327 | -6.9029322 | 6.57E-08 | 4.59E-06 | 8.17229942 | 2.06663614 | -2.850386 | 2.27E-58 | 2.20E-56 |
| CASC16 | 1.6054043 | 4.28898441 | 0.0001447 | 0.00099838 | 0.68495261 | 0.03508822 | 3.96838184 | 1.16E-06 | 1.96E-06 |
| CCND2-AS1 | -2.1172069 | -5.7176082 | 2.14E-06 | 5.44E-05 | 4.77214077 | 0.22258456 | -1.7581364 | 3.52E-35 | 3.87E-34 |
| CHL1-AS2 | -3.1847063 | -9.1277934 | 1.37E-10 | 4.46E-08 | 14.1873239 | 0.60842571 | -2.995167 | 7.72E-60 | 9.44E-58 |
| CNTFR-AS1 | 1.08708978 | 3.81130079 | 0.00056619 | 0.00269576 | -0.6220505 | 0.25949452 | -2.6486919 | 1.76E-52 | 8.71E-51 |
| DISC1FP1 | -1.6257141 | -4.1886327 | 0.00019334 | 0.00123782 | 0.40635147 | 0.03168309 | -1.2597242 | 9.30E-22 | 4.21E-21 |
| DNAH17-AS1 | 1.95314321 | 4.86563218 | 2.68E-05 | 0.00030396 | 2.31547085 | 0.00473663 | 3.00201638 | 1.01E-10 | 2.26E-10 |
| DNM3-IT1 | -1.1680858 | -3.621242 | 0.00096209 | 0.00407617 | -1.1262607 | 0.0284403 | 1.60192858 | 5.22E-06 | 8.38E-06 |
| DSCAM-AS1 | 1.70093076 | 5.59929274 | 3.04E-06 | 7.07E-05 | 4.42991833 | 0.17194631 | 5.77395666 | 8.05E-24 | 4.15E-23 |
| DSCR9 | 2.04432407 | 6.29316495 | 3.90E-07 | 1.66E-05 | 6.4325936 | 0.04579268 | 1.07133597 | 1.11E-12 | 2.82E-12 |
| EIF1B-AS1 | -2.7127812 | -5.9391764 | 1.11E-06 | 3.47E-05 | 5.41257591 | 0.36031833 | -1.0308655 | 3.25E-52 | 1.55E-50 |
| EIPR1-IT1 | 1.6157028 | 5.16032756 | 1.12E-05 | 0.0001757 | 3.1618838 | 0.08165611 | 2.05784977 | 5.74E-21 | 2.45E-20 |
| ELF3-AS1 | 1.06590736 | 3.49268698 | 0.00137019 | 0.00535041 | -1.4610917 | 0.66651457 | 1.25209053 | 6.92E-32 | 6.10E-31 |
| EMX2OS | -1.124319 | -4.1935822 | 0.0001906 | 0.00122526 | 0.42004781 | 1.11975033 | -2.1564864 | 1.01E-42 | 1.92E-41 |
| FAM181A-AS1 | 1.2544536 | 4.2818768 | 0.00014771 | 0.00100936 | 0.66515918 | 0.0213509 | -1.6856544 | 3.08E-28 | 2.10E-27 |
| FAM41C | -1.8747067 | -3.2580402 | 0.00258173 | 0.00865005 | -2.0575884 | 0.03648678 | -1.4185712 | 1.91E-16 | 6.06E-16 |
| FAM83C-AS1 | 1.66785626 | 4.24948602 | 0.00016221 | 0.00108547 | 0.57507198 | 0.02923047 | 2.52275487 | 1.88E-23 | 9.52E-23 |
| FEZF1-AS1 | 1.19455259 | 2.87609447 | 0.00696707 | 0.01864885 | -2.9812293 | 0.01254701 | 2.896652 | 0.0031299 | 0.00399998 |
| FGF13-AS1 | -3.7989825 | -7.5637896 | 9.90E-09 | 1.18E-06 | 10.0206869 | 0.82812008 | -2.7835286 | 1.83E-57 | 1.58E-55 |
| FGF14-AS2 | -1.2061892 | -3.1504316 | 0.00343255 | 0.0107833 | -2.3241442 | 4.59054453 | -1.9302967 | 1.31E-55 | 8.62E-54 |
| FLG-AS1 | -1.1442565 | -3.3547406 | 0.00199243 | 0.00711478 | -1.8141942 | 0.19462486 | -1.4075338 | 9.37E-41 | 1.58E-39 |
| FOXD3-AS1 | 2.22772476 | 3.89448085 | 0.00044782 | 0.00226459 | -0.3982524 | 0.10160609 | 4.45761693 | 2.58E-34 | 2.71E-33 |
| GAS1RR | -2.1460568 | -7.1325573 | 3.39E-08 | 2.85E-06 | 8.81954286 | 0.99255076 | -2.2384346 | 5.99E-56 | 4.17E-54 |
| GRIK1-AS1 | -2.3002919 | -4.7341599 | 3.94E-05 | 0.00039657 | 1.94012972 | 1.73472693 | -1.653386 | 2.17E-22 | 1.02E-21 |
| GSEC | 2.20829654 | 4.4115748 | 0.00010137 | 0.00077615 | 1.02770527 | 0.39690184 | 1.40620637 | 3.42E-42 | 6.31E-41 |
| HAGLROS | 1.5174169 | 5.98179378 | 9.78E-07 | 3.21E-05 | 5.53563106 | 0.17981593 | 2.35697153 | 3.00E-23 | 1.50E-22 |
| HCG21 | 1.26932031 | 3.6855118 | 0.00080494 | 0.00356495 | -0.9569293 | 0.06655647 | -1.222026 | 4.31E-10 | 9.26E-10 |
| HHIP-AS1 | -2.8443199 | -8.2247534 | 1.56E-09 | 3.64E-07 | 11.8201688 | 0.42136225 | -1.0066229 | 2.72E-33 | 2.65E-32 |
| HLA-F-AS1 | -1.5680318 | -5.9128503 | 1.20E-06 | 3.59E-05 | 5.3365349 | 0.78894427 | -1.0498008 | 1.31E-37 | 1.73E-36 |
| HOTAIRM1 | -3.0236908 | -6.6164859 | 1.51E-07 | 8.79E-06 | 7.35842906 | 4.82276832 | -1.5331441 | 1.47E-43 | 3.00E-42 |
| HOXA-AS2 | -1.9643089 | -8.4517461 | 8.40E-10 | 2.21E-07 | 12.4254806 | 1.05746405 | -1.5450972 | 3.12E-49 | 1.10E-47 |
| HOXA-AS3 | 1.68591745 | 4.71235183 | 4.20E-05 | 0.00041563 | 1.87803947 | 0.2488348 | -1.8293406 | 2.63E-51 | 1.12E-49 |
| HOXA11-AS | 1.06386721 | 3.18034248 | 0.00317247 | 0.01016232 | -2.2505222 | 0.07701029 | 1.42946689 | 3.89E-05 | 5.85E-05 |
| HOXB-AS1 | -1.197554 | -3.3607971 | 0.00196017 | 0.00703959 | -1.7988336 | 4.5521714 | -1.2487983 | 1.09E-22 | 5.26E-22 |
| HOXB-AS4 | 1.12422777 | 2.94779882 | 0.0058058 | 0.01629962 | -2.8127846 | 0.015551 | 2.909685 | 2.14E-06 | 3.53E-06 |
| KCNJ2-AS1 | -2.0923855 | -6.569963 | 1.73E-07 | 9.73E-06 | 7.22563955 | 0.80576353 | -2.1061943 | 3.62E-56 | 2.64E-54 |
| LEF1-AS1 | 1.41876597 | 5.00858245 | 1.75E-05 | 0.00023532 | 2.72530084 | 0.01865023 | 2.34645116 | 2.40E-49 | 8.58E-48 |
| LGALS8-AS1 | 1.62554361 | 3.34185402 | 0.00206277 | 0.00730293 | -1.8468325 | 0.08891557 | 2.37717196 | 7.70E-40 | 1.21E-38 |
| LINC00052 | 1.71554981 | 3.00389736 | 0.0050271 | 0.01468744 | -2.6793289 | 0.06283937 | 5.65034849 | 1.93E-17 | 6.53E-17 |
| LINC00165 | -2.3018596 | -4.2897847 | 0.00014436 | 0.00099747 | 0.68718182 | 0.37568461 | -1.6714604 | 9.51E-32 | 8.29E-31 |
| LINC00337 | 2.36716351 | 5.5055189 | 4.01E-06 | 8.76E-05 | 4.1586985 | 0.03720066 | 2.74937876 | 2.30E-44 | 5.12E-43 |
| LINC00377 | -2.5273678 | -5.4731486 | 4.42E-06 | 9.07E-05 | 4.06509464 | 0.41902853 | -3.3324501 | 2.45E-76 | 1.50E-72 |
| LINC00484 | -1.9030581 | -3.6981474 | 0.00077712 | 0.00346687 | -0.9234938 | 0.27804086 | -2.1114241 | 1.95E-50 | 7.86E-49 |
| LINC00511 | 1.28461373 | 5.77772989 | 1.79E-06 | 4.68E-05 | 4.94600327 | 0.11284669 | 2.81490274 | 1.29E-30 | 1.02E-29 |
| LINC00514 | 1.23276815 | 4.30086173 | 0.00013981 | 0.00097565 | 0.71804884 | 0.02089623 | 2.88495353 | 4.12E-37 | 5.24E-36 |
| LINC00518 | 1.70817438 | 5.29249581 | 7.56E-06 | 0.00013425 | 3.54310017 | 0.01521725 | 3.99518479 | 0.00330705 | 0.00421932 |
| LINC00520 | 1.33138385 | 3.35546547 | 0.00198854 | 0.00710609 | -1.8123565 | 0.00889809 | 1.67647443 | 5.94E-07 | 1.02E-06 |
| LINC00607 | 1.62565166 | 6.32536426 | 3.55E-07 | 1.54E-05 | 6.52508627 | 0.07892856 | 1.78200426 | 6.18E-18 | 2.16E-17 |
| LINC00626 | 1.49823699 | 3.28126011 | 0.00242664 | 0.00823776 | -1.9994685 | 0.0175095 | 1.70773526 | 1.16E-05 | 1.81E-05 |
| LINC00632 | -1.0918059 | -2.4120678 | 0.02152093 | 0.04511173 | -4.0071369 | 0.30128892 | -1.3419351 | 7.01E-17 | 2.29E-16 |
| LINC00640 | -1.593895 | -5.7050621 | 2.22E-06 | 5.59E-05 | 4.73585476 | 0.58369946 | -2.4140142 | 8.65E-49 | 2.78E-47 |
| LINC00645 | 1.17632578 | 3.86598539 | 0.00048536 | 0.0023998 | -0.4751216 | 0.10336751 | -2.9995425 | 3.87E-23 | 1.93E-22 |
| LINC00665 | -1.6535882 | -4.4604213 | 8.79E-05 | 0.00070545 | 1.16495041 | 2.95633357 | 1.04199246 | 1.49E-31 | 1.28E-30 |
| LINC00667 | -2.4109697 | -5.858127 | 1.41E-06 | 3.98E-05 | 5.17841579 | 7.38567129 | -1.0277045 | 4.48E-49 | 1.54E-47 |
| LINC00670 | 1.43813523 | 3.91512408 | 0.0004224 | 0.00216975 | -0.3424377 | 0.02145244 | -2.0737242 | 9.41E-30 | 7.02E-29 |
| LINC00866 | -1.5626193 | -3.5468455 | 0.00118117 | 0.00477257 | -1.3206815 | 0.10351458 | -1.1357933 | 3.64E-06 | 5.91E-06 |
| LINC00870 | 1.01701371 | 3.26797758 | 0.00251423 | 0.00847621 | -2.0327404 | 0.0168898 | 1.5797659 | 6.83E-10 | 1.45E-09 |
| LINC00891 | -1.2844551 | -2.4937083 | 0.017772 | 0.03858499 | -3.8353024 | 0.03887675 | -1.1717156 | 3.52E-23 | 1.76E-22 |
| LINC00920 | -3.1094213 | -5.9054814 | 1.23E-06 | 3.61E-05 | 5.31524718 | 1.59619408 | -1.5085518 | 1.68E-26 | 1.01E-25 |
| LINC00923 | 1.12600349 | 2.55042054 | 0.01553061 | 0.03478543 | -3.713649 | 0.03714717 | -1.3041424 | 3.03E-34 | 3.17E-33 |
| LINC00958 | -1.5681419 | -5.6251041 | 2.82E-06 | 6.76E-05 | 4.50457912 | 0.95469992 | -1.1155101 | 4.85E-27 | 3.04E-26 |
| LINC00987 | -2.7058377 | -3.9486507 | 0.00038409 | 0.0020132 | -0.2515658 | 2.25191414 | -1.3311037 | 2.95E-49 | 1.04E-47 |
| LINC01013 | -1.3077327 | -3.2703065 | 0.00249866 | 0.00842953 | -2.0269115 | 0.1318709 | -1.1024427 | 9.16E-16 | 2.78E-15 |
| LINC01028 | -1.8658935 | -5.28141 | 7.81E-06 | 0.00013774 | 3.51109802 | 0.57740133 | -3.981649 | 8.24E-70 | 8.41E-67 |
| LINC01060 | -2.6390747 | -6.4675022 | 2.34E-07 | 1.17E-05 | 6.93265157 | 0.31800868 | -1.3948619 | 2.40E-21 | 1.05E-20 |
| LINC01063 | 1.28321875 | 3.48916343 | 0.00138344 | 0.00538926 | -1.470193 | 0.22739805 | 1.40769171 | 3.92E-19 | 1.49E-18 |
| LINC01082 | 1.07193549 | 4.26887847 | 0.00015337 | 0.00103623 | 0.62898455 | 0.10623314 | -1.1639998 | 1.08E-40 | 1.80E-39 |
| LINC01088 | -1.2549263 | -3.2108445 | 0.00292664 | 0.0095311 | -2.175069 | 0.62759525 | -2.1489885 | 9.86E-31 | 7.85E-30 |
| LINC01091 | -2.8971351 | -7.7149842 | 6.46E-09 | 9.56E-07 | 10.4369607 | 0.16434698 | -1.5997118 | 8.76E-52 | 3.97E-50 |
| LINC01121 | 1.3572708 | 5.0290767 | 1.65E-05 | 0.00022645 | 2.78418133 | 0.05657921 | 1.016605 | 5.34E-09 | 1.07E-08 |
| LINC01136 | 1.0770112 | 4.86997431 | 2.64E-05 | 0.00030181 | 2.32789496 | 0.03662951 | 1.8815298 | 1.21E-27 | 7.96E-27 |
| LINC01140 | -1.7878349 | -5.1732429 | 1.08E-05 | 0.0001713 | 3.19910175 | 1.21890176 | -2.1699659 | 5.74E-50 | 2.18E-48 |
| LINC01163 | 1.05664567 | 3.46978628 | 0.0014586 | 0.00560161 | -1.5201692 | 0.00721055 | 1.46865546 | 1.66E-06 | 2.77E-06 |
| LINC01186 | -2.1159232 | -2.6236132 | 0.01302211 | 0.03043684 | -3.5539586 | 0.7248633 | -2.8374906 | 5.98E-55 | 3.55E-53 |
| LINC01198 | -4.8772487 | -5.1958855 | 1.01E-05 | 0.00016281 | 3.26436966 | 0.85244103 | -1.8071999 | 1.54E-32 | 1.41E-31 |
| LINC01208 | 1.62554257 | 2.52131195 | 0.0166464 | 0.03672885 | -3.7763203 | 0.07377187 | 3.25436777 | 0.0247649 | 0.02902441 |
| LINC01215 | 1.20123001 | 2.75667109 | 0.00939668 | 0.02366123 | -3.2562161 | 0.11725753 | 1.15501762 | 0.04163928 | 0.047884 |
| LINC01230 | 1.51529063 | 4.34509422 | 0.00012298 | 0.00088605 | 0.84151865 | 1.61491299 | -4.4133722 | 9.04E-48 | 2.67E-46 |
| LINC01235 | -3.3369933 | -5.5587932 | 3.43E-06 | 7.80E-05 | 4.31277506 | 5.67494682 | -1.4943535 | 2.30E-31 | 1.96E-30 |
| LINC01252 | -1.7392121 | -4.3603894 | 0.00011764 | 0.00086251 | 0.88429071 | 0.46066636 | -1.0377159 | 3.20E-20 | 1.30E-19 |
| LINC01273 | 1.25369364 | 4.70903137 | 4.24E-05 | 0.00041751 | 1.86859024 | 0.47374363 | -1.027381 | 9.74E-30 | 7.25E-29 |
| LINC01300 | 1.33643079 | 4.81722521 | 3.09E-05 | 0.00033291 | 2.17707945 | 0.03462479 | -1.4917941 | 3.12E-28 | 2.13E-27 |
| LINC01354 | -1.4388608 | -2.9385049 | 0.00594531 | 0.01656751 | -2.8347542 | 0.85486444 | -1.9362291 | 6.49E-51 | 2.66E-49 |
| LINC01359 | -1.171507 | -4.161368 | 0.00020912 | 0.00130807 | 0.33099038 | 0.33960743 | -1.0527807 | 2.45E-41 | 4.30E-40 |
| LINC01412 | -1.8513784 | -4.3209214 | 0.00013191 | 0.00092985 | 0.77400141 | 0.22295141 | -2.3614391 | 1.79E-44 | 4.00E-43 |
| LINC01505 | 1.37718071 | 3.17333613 | 0.00323166 | 0.01029117 | -2.2678004 | 0.01081829 | 2.72212084 | 1.69E-13 | 4.48E-13 |
| LINC01589 | -2.3258525 | -7.109918 | 3.62E-08 | 2.99E-06 | 8.75595193 | 1.0748765 | -2.3966068 | 9.55E-55 | 5.46E-53 |
| LINC01615 | 2.64670416 | 4.04856398 | 0.00028897 | 0.0016369 | 0.02081823 | 0.2011995 | 1.93566139 | 2.78E-27 | 1.78E-26 |
| LINC01625 | -1.6634212 | -2.9160297 | 0.00629583 | 0.01734639 | -2.8877156 | 0.3264743 | -2.7818077 | 1.20E-47 | 3.47E-46 |
| LINC01655 | 3.01645207 | 5.34223181 | 6.52E-06 | 0.00012065 | 3.68672646 | 0.05991752 | 2.06451464 | 1.47E-13 | 3.93E-13 |
| LINC01697 | -4.1343635 | -7.665471 | 7.43E-09 | 1.01E-06 | 10.3009307 | 1.24071003 | -3.9679284 | 9.63E-61 | 1.34E-58 |
| LINC01703 | -1.5166345 | -3.2474168 | 0.00265581 | 0.00883762 | -2.084109 | 0.65648956 | 1.11052074 | 3.40E-14 | 9.45E-14 |
| LINC01705 | 4.71216692 | 6.35208348 | 3.28E-07 | 1.50E-05 | 6.60179318 | 0.02325826 | 5.70061763 | 7.66E-56 | 5.15E-54 |
| LINC01727 | 1.02432852 | 3.82008089 | 0.00055238 | 0.00265069 | -0.5985128 | 0.00558819 | 2.25712104 | 7.39E-13 | 1.90E-12 |
| LINC01747 | 1.48255037 | 6.92047225 | 6.25E-08 | 4.45E-06 | 8.22191091 | 0.00664223 | 1.75989929 | 3.51E-22 | 1.63E-21 |
| LINC01778 | -3.4356105 | -8.1662347 | 1.84E-09 | 3.90E-07 | 11.6630357 | 0.4571418 | -1.0141563 | 3.72E-29 | 2.67E-28 |
| LINC01797 | 1.70142114 | 4.33795786 | 0.00012555 | 0.0008969 | 0.82157569 | 0.03893713 | -1.0779913 | 8.48E-15 | 2.43E-14 |
| LINC01798 | 1.02429239 | 3.91104585 | 0.00042731 | 0.00218522 | -0.3534728 | 0.06966036 | -1.0106746 | 1.95E-22 | 9.23E-22 |
| LINC01819 | -3.7534399 | -6.7057573 | 1.17E-07 | 7.12E-06 | 7.6127819 | 3.05888044 | -1.1477669 | 1.09E-25 | 6.26E-25 |
| LINC01852 | -1.4382735 | -4.5500458 | 6.77E-05 | 0.00058713 | 1.41768051 | 0.73160783 | -1.0796482 | 1.08E-30 | 8.63E-30 |
| LINC01874 | -3.1753838 | -9.8880127 | 1.91E-11 | 8.50E-09 | 16.0926874 | 0.14435876 | -1.2518961 | 4.71E-20 | 1.89E-19 |
| LINC01929 | 3.9896398 | 4.84303359 | 2.86E-05 | 0.00031826 | 2.25083676 | 0.05520327 | 3.5865758 | 6.14E-39 | 8.90E-38 |
| LINC01978 | 1.50983189 | 3.58895686 | 0.00105186 | 0.00436561 | -1.210846 | 0.03101226 | 2.09797814 | 9.45E-22 | 4.27E-21 |
| LINC01985 | -3.7653184 | -7.9083205 | 3.76E-09 | 6.12E-07 | 10.9653301 | 0.789011 | -3.1666073 | 5.97E-63 | 1.30E-60 |
| LINC02014 | -1.9664067 | -5.2108095 | 9.63E-06 | 0.00015959 | 3.3074015 | 0.15320104 | 1.1406846 | 0.01800711 | 0.02139109 |
| LINC02022 | 1.19404858 | 3.28979495 | 0.00237191 | 0.0080744 | -1.9780534 | 0.07505365 | -1.9111086 | 7.95E-40 | 1.25E-38 |
| LINC02104 | -1.2488817 | -2.819577 | 0.00803249 | 0.02084551 | -3.1122524 | 0.92837992 | -2.3321562 | 6.16E-35 | 6.68E-34 |
| LINC02202 | -3.3088012 | -6.8961924 | 6.70E-08 | 4.61E-06 | 8.15322858 | 1.49043535 | -3.0175443 | 5.22E-62 | 8.87E-60 |
| LINC02245 | -1.2776347 | -2.9661487 | 0.00553937 | 0.01569594 | -2.7692901 | 0.01044581 | 1.35496381 | 1.64E-05 | 2.53E-05 |
| LINC02257 | 1.60282974 | 7.1877053 | 2.89E-08 | 2.52E-06 | 8.97423448 | 0.06919657 | 2.07700297 | 7.49E-18 | 2.60E-17 |
| LINC02280 | 2.19044737 | 5.04060208 | 1.59E-05 | 0.00022134 | 2.81730638 | 0.01656842 | 1.986941 | 1.53E-10 | 3.37E-10 |
| LINC02347 | 1.16289635 | 4.10922996 | 0.0002429 | 0.00145057 | 0.18729594 | 0.01813599 | -2.2287144 | 1.47E-35 | 1.64E-34 |
| LINC02356 | -1.3484815 | -3.4103124 | 0.0017146 | 0.00634531 | -1.6727533 | 0.4864797 | -1.1986797 | 5.41E-27 | 3.38E-26 |
| LINC02511 | -4.0706969 | -7.2102406 | 2.71E-08 | 2.45E-06 | 9.0373589 | 0.67283309 | -4.0116803 | 1.64E-66 | 7.15E-64 |
| LINC02515 | -2.9821992 | -4.830818 | 2.97E-05 | 0.00032403 | 2.21591823 | 2.14375341 | -1.6052186 | 2.65E-31 | 2.24E-30 |
| LINC02516 | 1.2082664 | 3.46716305 | 0.00146907 | 0.00563739 | -1.5269251 | 0.05554916 | 1.41442982 | 0.00163486 | 0.00214212 |
| LINC02518 | -1.705621 | -2.5770348 | 0.01457105 | 0.03307601 | -3.6559282 | 0.46192912 | -1.8315176 | 2.83E-36 | 3.35E-35 |
| LINC02532 | 1.17601187 | 4.24679568 | 0.00016348 | 0.00108978 | 0.56759807 | 0.02373912 | 2.28988102 | 0.00064362 | 0.00087285 |
| LINC02544 | 2.02842113 | 2.90109973 | 0.00653933 | 0.0178163 | -2.9227652 | 0.44895257 | 3.57383339 | 5.29E-49 | 1.79E-47 |
| LINC02574 | 2.00021681 | 3.51721989 | 0.00128121 | 0.00509252 | -1.3976082 | 0.04573914 | 1.72309565 | 1.72E-09 | 3.58E-09 |
| LINC02577 | 1.50887835 | 4.42882713 | 9.64E-05 | 0.00074749 | 1.07613772 | 0.00513746 | 3.78925044 | 5.68E-05 | 8.40E-05 |
| LINC02580 | -2.1582617 | -4.6791284 | 4.63E-05 | 0.00044652 | 1.78354917 | 0.87410273 | -2.2003936 | 1.11E-55 | 7.38E-54 |
| LINC02586 | -2.3016339 | -5.6122392 | 2.92E-06 | 6.87E-05 | 4.46736653 | 0.35551474 | -1.0224387 | 3.63E-23 | 1.81E-22 |
| LINC02593 | 1.39360408 | 3.98551517 | 0.00034588 | 0.0018642 | -0.1513348 | 0.30686459 | 2.19897068 | 5.36E-08 | 9.97E-08 |
| LRRC8C-DT | -1.2685463 | -5.1172536 | 1.27E-05 | 0.00018757 | 3.03782054 | 0.775683 | -1.4601532 | 4.68E-50 | 1.81E-48 |
| LUARIS | -5.485461 | -10.299391 | 6.81E-12 | 4.75E-09 | 17.0897012 | 0.44657168 | -3.2345889 | 4.39E-43 | 8.63E-42 |
| LYPLAL1-DT | 1.21511092 | 4.05247709 | 0.00028575 | 0.00162693 | 0.03153233 | 0.55342442 | -1.3815189 | 2.93E-43 | 5.82E-42 |
| MAFA-AS1 | 2.20668894 | 3.89763978 | 0.00044383 | 0.0022491 | -0.3897183 | 0.0187042 | 4.25664963 | 4.12E-24 | 2.17E-23 |
| MAFG-DT | 1.79220902 | 5.26614111 | 8.17E-06 | 0.00014309 | 3.46702772 | 1.5179144 | 1.62784855 | 3.18E-41 | 5.51E-40 |
| MAFTRR | -1.2752862 | -4.4851702 | 8.18E-05 | 0.00066818 | 1.23462461 | 0.3045401 | -1.952155 | 6.67E-49 | 2.21E-47 |
| MAGI2-AS3 | -1.8759864 | -6.3530862 | 3.27E-07 | 1.50E-05 | 6.60467108 | 3.9632938 | -2.1170707 | 2.01E-62 | 3.84E-60 |
| MAPT-IT1 | 1.66016511 | 4.8910525 | 2.48E-05 | 0.00029217 | 2.38822909 | 0.08141802 | 2.22952233 | 0.00079077 | 0.00106472 |
| MEF2C-AS1 | -1.3251714 | -5.4521116 | 4.70E-06 | 9.49E-05 | 4.00427101 | 0.21263259 | -2.0847962 | 5.48E-59 | 5.99E-57 |
| MIR133A1HG | 1.27281778 | 3.7619365 | 0.0006503 | 0.00300294 | -0.7539974 | 0.24501269 | -1.5430456 | 0.015124 | 0.01811386 |
| MIR193BHG | -1.0307399 | -2.3802471 | 0.02316761 | 0.0477728 | -4.0730329 | 2.32784795 | -1.0000122 | 9.33E-13 | 2.38E-12 |
| MIR22HG | -1.358708 | -6.5231784 | 1.99E-07 | 1.07E-05 | 7.09194746 | 11.7439845 | -1.5052584 | 5.49E-43 | 1.06E-41 |
| MIR99AHG | -2.3269813 | -4.6081695 | 5.71E-05 | 0.00051823 | 1.58216735 | 2.21471381 | -1.8406504 | 2.46E-60 | 3.34E-58 |
| MNX1-AS2 | 1.14270641 | 2.52009647 | 0.01669456 | 0.03681847 | -3.7789267 | 0.13468407 | 2.26921602 | 3.32E-08 | 6.27E-08 |
| NARF-AS1 | 1.29277356 | 4.71599767 | 4.16E-05 | 0.00041264 | 1.88841604 | 0.0425452 | 1.61472132 | 5.66E-18 | 1.99E-17 |
| NKAIN3-IT1 | -3.1280332 | -5.2003609 | 9.93E-06 | 0.00016121 | 3.27727294 | 0.13880741 | -1.2745303 | 1.88E-30 | 1.47E-29 |
| NRIR | 1.01487794 | 2.62534702 | 0.0129675 | 0.03036733 | -3.5501398 | 0.18591158 | 1.01340401 | 0.00272349 | 0.00350176 |
| OVOL1-AS1 | 1.36784833 | 4.07886884 | 0.00026498 | 0.00154834 | 0.10388073 | 0.08834072 | 1.13382526 | 1.31E-09 | 2.74E-09 |
| OXCT1-AS1 | 1.21907611 | 4.94118318 | 2.14E-05 | 0.00026403 | 2.53187175 | 0.59021429 | -2.1773254 | 1.99E-58 | 1.97E-56 |
| PCAT19 | -1.3955826 | -3.1472994 | 0.00346093 | 0.01086546 | -2.3318322 | 3.55347672 | -1.6081591 | 1.57E-52 | 7.82E-51 |
| PGM5-AS1 | -4.0731387 | -10.307315 | 6.68E-12 | 4.75E-09 | 17.1086724 | 1.55534028 | -3.3951416 | 7.92E-64 | 2.11E-61 |
| PGM5P3-AS1 | -3.1617845 | -10.130856 | 1.04E-11 | 5.63E-09 | 16.6841365 | 0.23522378 | -3.5557564 | 3.93E-66 | 1.41E-63 |
| PGM5P4-AS1 | -2.2835899 | -7.3294456 | 1.93E-08 | 1.91E-06 | 9.3704006 | 0.21054269 | -3.412422 | 1.72E-59 | 1.99E-57 |
| PVT1 | -1.2494295 | -3.7620769 | 0.00065004 | 0.00300294 | -0.7536231 | 1.52396211 | 1.34313974 | 7.51E-25 | 4.07E-24 |
| RAPGEF4-AS1 | 1.53524825 | 4.539014 | 6.99E-05 | 0.00059959 | 1.38651155 | 0.02813633 | -1.6324715 | 1.37E-35 | 1.53E-34 |
| RBMS3-AS3 | -2.7298364 | -5.4975162 | 4.11E-06 | 8.85E-05 | 4.13555608 | 0.98907356 | -2.5491792 | 9.70E-59 | 9.89E-57 |
| RBPMS-AS1 | -2.1796283 | -5.9664319 | 1.02E-06 | 3.31E-05 | 5.49128089 | 2.22549874 | -1.9579056 | 6.36E-52 | 2.95E-50 |
| RHOXF1-AS1 | -4.9922361 | -7.2733728 | 2.26E-08 | 2.13E-06 | 9.2139249 | 3.75042715 | -3.0242829 | 7.14E-57 | 5.69E-55 |
| RNF139-AS1 | 1.45230897 | 4.85884418 | 2.73E-05 | 0.00030725 | 2.29605175 | 0.26159976 | 1.16927349 | 1.31E-20 | 5.48E-20 |
| ROCR | -3.4789547 | -2.9615429 | 0.00560514 | 0.01579982 | -2.7802218 | 12.7126868 | -1.3849714 | 5.10E-28 | 3.42E-27 |
| RUNDC3A-AS1 | 1.32957254 | 5.09527215 | 1.36E-05 | 0.00019605 | 2.97454764 | 0.06307424 | 2.14770556 | 1.00E-18 | 3.69E-18 |
| SACS-AS1 | -2.4641404 | -5.8923169 | 1.27E-06 | 3.71E-05 | 5.27721347 | 0.16303332 | -3.0734696 | 9.06E-60 | 1.09E-57 |
| SEMA6A-AS2 | -1.586414 | -4.9782002 | 1.92E-05 | 0.00024666 | 2.63806558 | 0.23617887 | -1.2045015 | 7.38E-25 | 4.01E-24 |
| SH3TC2-DT | 1.03512654 | 3.8564233 | 0.00049864 | 0.00245054 | -0.5008698 | 0.02217432 | -1.4627493 | 1.09E-35 | 1.23E-34 |
| SLC12A5-AS1 | 1.16060148 | 2.89590976 | 0.00662603 | 0.01801233 | -2.9349244 | 0.02178934 | 3.0898132 | 4.46E-30 | 3.40E-29 |
| SLC14A2-AS1 | -3.2673674 | -6.0539611 | 7.90E-07 | 2.76E-05 | 5.743877 | 0.23969398 | -1.8167122 | 7.08E-27 | 4.38E-26 |
| SNHG25 | 1.36812526 | 3.70327992 | 0.00076608 | 0.00343018 | -0.9098991 | 0.6432372 | 1.81111167 | 2.24E-24 | 1.19E-23 |
| SNHG26 | -3.8498213 | -7.8199158 | 4.81E-09 | 7.58E-07 | 10.7242862 | 1.64716249 | -2.0847738 | 5.42E-51 | 2.24E-49 |
| SOX9-AS1 | -1.3425299 | -3.2416359 | 0.00269697 | 0.00895627 | -2.098522 | 1.4549456 | -1.3774082 | 1.09E-26 | 6.66E-26 |
| TDRG1 | 2.03698117 | 5.91530528 | 1.19E-06 | 3.59E-05 | 5.34362679 | 0.00877334 | 2.39335286 | 7.21E-13 | 1.85E-12 |
| TDRKH-AS1 | -1.4092375 | -3.2193016 | 0.00286177 | 0.00937608 | -2.1540824 | 0.37422788 | 1.31518215 | 4.09E-36 | 4.77E-35 |
| THRB-AS1 | -2.880455 | -4.8969748 | 2.44E-05 | 0.00028815 | 2.40518802 | 0.09951715 | -1.0637778 | 1.66E-42 | 3.10E-41 |
| TLR8-AS1 | 1.2296426 | 3.2071635 | 0.00295532 | 0.00958614 | -2.1841948 | 0.00885566 | 2.29199843 | 2.49E-05 | 3.80E-05 |
| TPRG1-AS1 | -2.1154585 | -4.3415677 | 0.00012424 | 0.00089168 | 0.83166263 | 2.24761505 | -1.4818274 | 1.32E-40 | 2.20E-39 |
| TRHDE-AS1 | -2.0647617 | -5.2315971 | 9.05E-06 | 0.00015252 | 3.36735697 | 2.89974641 | -5.1958848 | 3.12E-55 | 1.91E-53 |
| TRPM2-AS | -1.067113 | -2.6926866 | 0.01100351 | 0.02680905 | -3.4005647 | 0.75521793 | 1.26998924 | 8.28E-09 | 1.64E-08 |
| U62317.3 | 1.23446744 | 3.83993916 | 0.00052235 | 0.0025517 | -0.5452015 | 0.17884264 | 1.0034016 | 1.48E-11 | 3.48E-11 |
| VAC14-AS1 | 1.05739117 | 3.35882331 | 0.00197063 | 0.00705757 | -1.8038411 | 0.01871726 | 1.19166561 | 3.24E-06 | 5.29E-06 |
| WARS2-IT1 | -2.1384634 | -3.7482673 | 0.00067566 | 0.00310199 | -0.7904155 | 0.34705597 | -1.163172 | 2.91E-48 | 8.92E-47 |
| WASIR2 | 1.28546119 | 3.82276422 | 0.00054823 | 0.00263853 | -0.5913153 | 0.29744989 | 1.74432489 | 1.28E-17 | 4.39E-17 |
| WDFY3-AS2 | -2.2396895 | -5.4373395 | 4.92E-06 | 9.80E-05 | 3.96156534 | 1.09986698 | -1.4214777 | 1.26E-56 | 9.62E-55 |
| WDR86-AS1 | -1.206722 | -4.1449301 | 0.00021924 | 0.00135397 | 0.28562695 | 2.8156495 | -2.3318776 | 1.15E-57 | 1.04E-55 |
| WWC2-AS2 | -1.3325633 | -3.0827868 | 0.00409749 | 0.01241702 | -2.489263 | 0.41342623 | -1.0084597 | 2.19E-34 | 2.31E-33 |
| Z97989.1 | -1.6173631 | -3.7868805 | 0.00060639 | 0.00285375 | -0.6874071 | 1.14701784 | -1.5569807 | 2.47E-53 | 1.32E-51 |
| ZFHX4-AS1 | -1.269983 | -2.958721 | 0.00564579 | 0.01589608 | -2.7869146 | 0.46643601 | -1.042736 | 4.36E-21 | 1.87E-20 |
